# Anti-virulence and bactericidal activities of Stattic against *Shigella sonnei*

**DOI:** 10.1128/aem.01074-23

**Published:** 2023-11-30

**Authors:** Mingfang Wang, Jia Zeng, Huihui Tan, Quan Guo, Xia Li, Xiwen Ling, Jinyue Zhang, Shihao Song, Yinyue Deng

**Affiliations:** 1 School of Pharmaceutical Sciences (Shenzhen), Shenzhen Campus of Sun Yat-sen University, Sun Yat-sen University, Shenzhen, China; 2 School of Pharmaceutical Sciences, Hainan University, Haikou, China; Centers for Disease Control and Prevention, Atlanta, Georgia, USA

**Keywords:** *Shigella sonnei*, Stattic, GalU, antibiotic, virulence

## Abstract

*Shigella sonnei* is an important human pathogen that provokes severe colitis and diarrhea, causing high mortality and morbidity worldwide. Because of increasing drug resistance, it is particularly important to develop new antibacterial agents against *S. sonnei*. In this study, we report that Stattic, a potent signal transducer and activator of transcription 3 inhibitor, was found to be effective against *S. sonnei*. Our results showed that Stattic has dual antibacterial mechanisms. At low concentrations, Stattic binds to GalU of *S. sonnei*, inhibits its enzyme activity, and then reduces biofilm formation and extracellular polysaccharide production, which ultimately attenuates *S. sonnei* virulence in human cell lines. At high concentrations, Stattic could kill bacteria by disrupting the integrity of the bacterial cell membrane, causing space collapse and the outflow of contents. Furthermore, Stattic showed excellent synergistic effects with antibiotics against *S. sonnei*. In addition, Stattic also showed anti-virulence activity against *Escherichia coli* and *Klebsiella pneumoniae*. Together, our findings suggest the promising potential and advantages of Stattic as a new therapeutic agent against bacterial pathogens.

**IMPORTANCE:**

*Shigella sonnei* is a major human enteric pathogen that causes bacillary dysentery. The increasing spread of drug-resistant *S. sonnei* strains has caused an emergent need for the development of new antimicrobial agents against this pathogenic bacterium. In this study, we demonstrate that Stattic employs two antibacterial mechanisms against *S. sonnei*. It exerted both anti-virulence activity and bactericidal activity against *S. sonnei*, suggesting that it shows advantages over traditional antibiotics. Moreover, Stattic showed excellent synergistic effects with kanamycin, ampicillin, chloramphenicol, and gentamicin against *S. sonnei*. Our findings suggest that Stattic has promising potential for development as a new antibiotic or as an adjuvant to antibiotics for infections caused by *S. sonnei*.

## INTRODUCTION

*Shigella* is a bacterial pathogen that leads to diarrhea and is an important cause of mortality and morbidity worldwide (1, 2). It is highly susceptible and easily transmitted through direct human-to-human contact or contaminated water and food, and disease can be caused by low infectious doses of approximately 10–100 cells (3). Based on biochemical and serological tests, *Shigella* is classified into the following four subgroups: *Shigella dysenteriae* (*S. dysenteriae*), *Shigella flexneri* (*S. flexneri*), *Shigella boydii* (*S. boydii*), and *Shigella sonnei* (*S. sonnei*) (4–6). At present, the reported cases of *Shigella* infection are mostly caused by *S. flexneri* and *S. sonnei* (7–10). A previous study demonstrated that the Type VI Secretion System (T6SS) encoded by *S. sonnei* gives it a competitive advantage over *S. flexneri* and *Escherichia coli* lacking T6SS in the intestinal tract (11). A grim reality is that due to the abuse of antibiotics, *S. sonnei* has developed serious antibiotic resistance (12). Therefore, there is an urgent need to develop new antibiotics or find another treatment against *S. sonnei* (13).

The mechanisms of bacterial resistance to antibiotics are varied, among which the formation of biofilms is one of the most effective methods (14, 15). Bacterial biofilms are characterized by adhesion to the solid surface and the production of an extracellular polymer matrix composed of extracellular polysaccharides (EPS), proteins, DNA, and lipids (16–19). EPS not only provides bacteria with adhesion but also protects bacteria from the host immune response and antibacterial therapy (20). In addition, EPS can create a pathogenic environment (such as acidic pH and hypoxia) and promote the release of key virulence factors (21). The *galU* gene encodes a highly conserved protein (known as GalU) that catalyzes the synthesis of UDP-α-D-glucose, which is involved in the lipopolysaccharide (LPS) core region biosynthetic process in *Enterobacteriaceae* (22–24). Besides, UDP-α-D-glucose is the important intermediate in several different metabolic pathways and biosynthetic reactions involved in the biosynthesis of capsular polysaccharides (CPS) and EPS in bacteria (25–27). Previous studies revealed that GalU is critical for bacterial attachment, biofilm formation, drug resistance, and immune evasion (28–31). Mutation of *galU* caused reduced virulence in many gram-negative pathogens, suggesting the vital role of GalU in the pathogenesis of various bacterial pathogens (32–34).

Due to the importance of GalU in the physiological processes and pathogenesis of bacteria, it has attracted attention to be used as a target for the development of antibacterial drugs (24, 35, 36). It was found that pyrimidinyl benzamide could inhibit GalU to attenuate *Listeria* virulence (35), and some compounds target GalU and reduce *Streptococcus* virulence (36). In this study, we discovered that Stattic, which is a small molecule that selectively inhibits the activation of signal transducer and activator of transcription 3 (STAT3) by inhibiting phosphorylation and dimerization processes (37, 38), displayed strong antimicrobial activity against *S. sonnei* by inhibiting virulence through targeting GalU or killing bacterial cells directly. Our findings suggest that Stattic might have an advantage over conventional antibiotics for the treatment of *S. sonnei* infections.

## RESULTS

### Screening of leading compounds that inhibit the virulence of *S. sonnei*

**Fig 1:**
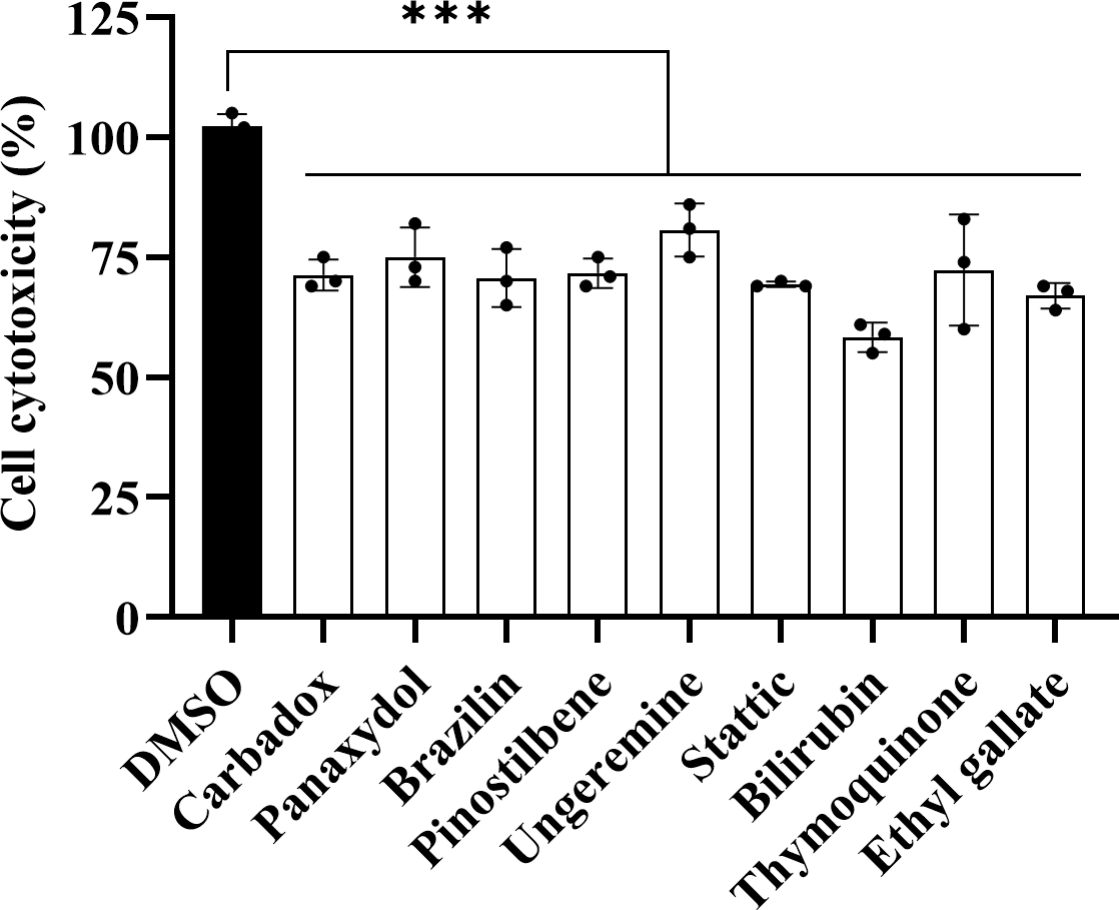
Effects of compounds on the virulence of *S. sonnei*. All compounds were dissolved in Dimethyl sulfoxide (DMSO). LDH release was used to assess cytotoxicity, and the amount of LDH released by *S. sonnei* infectious assays treated with the same volume of DMSO was used as a control and defined as 100%, aimed to normalize the LDH production of HeLa cells infected by *S. sonnei* in the presence of compounds. The data are presented as the mean ± SD (*n* = 3, independent measurements). One-way ANOVA was used to determine the significance of the results (*P < 0.05; **P < 0.01; ***P < 0.001; ns = no significance).

Biofilm development offers a protected mode of growth that not only permits cells to survive in adverse environments but also allows microorganisms from microbial clusters to colonize new niches, which are a major source of bacterial infection (39). Biofilm formation is also a key pathogenic phenotype of *S. sonnei* (40). We first evaluated the inhibitory effects of approximately 1,000 compounds on *S. sonnei* biofilm formation at a final concentration of 20 µM to screen for leading compounds against *S. sonnei*, and 42 candidate compounds were selected because they could effectively decrease more than 15% biofilm production (Fig. S1). Then, we used the HeLa cells infection model to detect whether these compounds had an effect on the virulence of *S. sonnei*, which was evaluated by the content of lactate dehydrogenase (LDH) in the supernatant of the culture medium when cells were co-incubated with *S. sonnei*. The results showed that among these selected 42 compounds screened with biofilm formation assays, only nine compounds could attenuate *S. sonnei* virulence by more than 15% (Fig. 1), among which Bilirubin, Stattic, and Ethyl gallate had significant inhibitory activity, lowering *S. sonnei* virulence by more than 30% (Fig. 1).

### Stattic can bind to GalU

**Fig 2:**
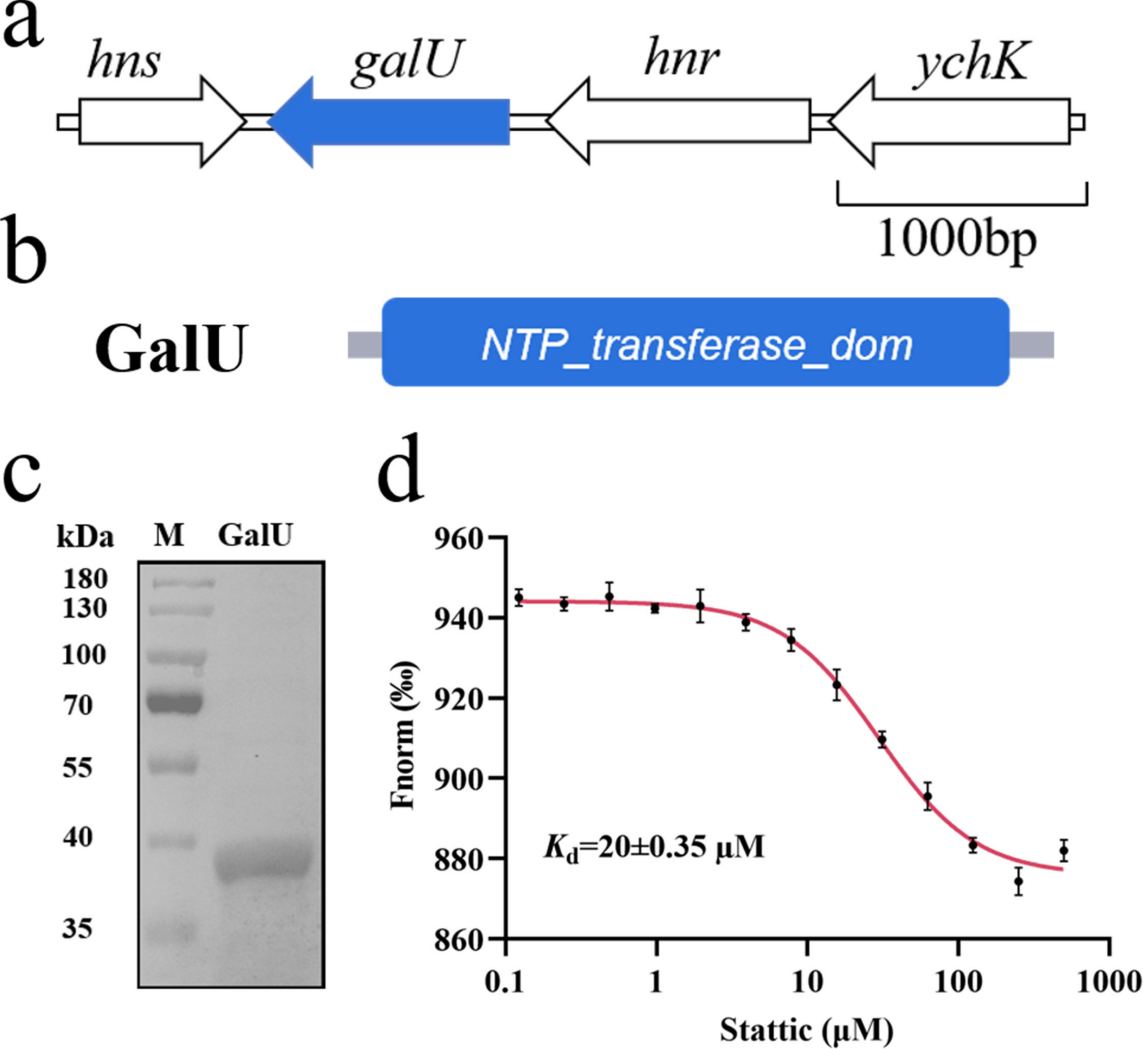
Stattic binds to GalU. (**a**) Genomic organization of the *galU* region in *S. sonnei* Ss046. (**b**) Analysis of the domain structure of GalU. (**c**) SDS‒PAGE analysis of the GalU protein. (**d**) MST analysis of the binding of Stattic to GalU.

As GalU plays a key role in the biofilm formation and pathogenesis of bacterial pathogens, we continued to investigate whether there is a relationship between these compounds and GalU in *S. sonnei*. The gene *galU* is located between *hns* and *hnr*, and the homologous protein of GalU in *S. sonnei* shares 100% identity with that in *E. coli*, based on the National Center for Biotechnology Information Basic Local Alignment Search Tool (BLAST) program, which contains a nucleotide transferase domain (Fig. 2a and b). Molecular docking is a common tool for predicting and understanding the binding of small molecules to proteins, and this method was performed by the AutoDock program (41, 42). The docking results showed that Brazilin, Ungeremine, Stattic, and Bilirubin had a strong affinity for GalU, and their binding energies were less than −6 kcal/mol (Table S1). We then used microscale thermophoresis (MST), which is a novel biomolecule interaction analysis technique that measures ligand-target molecule affinity by observing changes in the target molecule’s conformational size, charge, and solvation state after the ligand binds to the target molecule (43), to confirm the interaction between these compounds and GalU, which was purified using affinity chromatography (Fig. 2c). Surprisingly, MST experiments revealed that only Stattic could bind to GalU with a well-fit curve, and the dissociation constant (*K*_d_) obtained by MST was 20 ± 0.35 µM (Fig. 2d), while the remaining eight compounds could not bind to GalU (Fig. S2). GalU is highly conserved in gram-negative bacteria. We then constructed and expressed GalU homolog proteins from four other species, including *Klebsiella oxytoca*, *Acinetobacter baumannii*, *Burkholderia cenocepacia*, and *Pseudomonas aeruginosa*, which share 94%, 58%, 54%, and 40% identity with GalU of *S. sonnei*, and tested their binding ability to Stattic (Fig. S3a). The results showed that the GalU protein of *K. oxytoca* also bound to Stattic with a *K*_d_ of 50 ± 0.25 µM (Fig. S3b through e).

### Glutamine 109 is the key site for binding and activity

**Fig 3:**
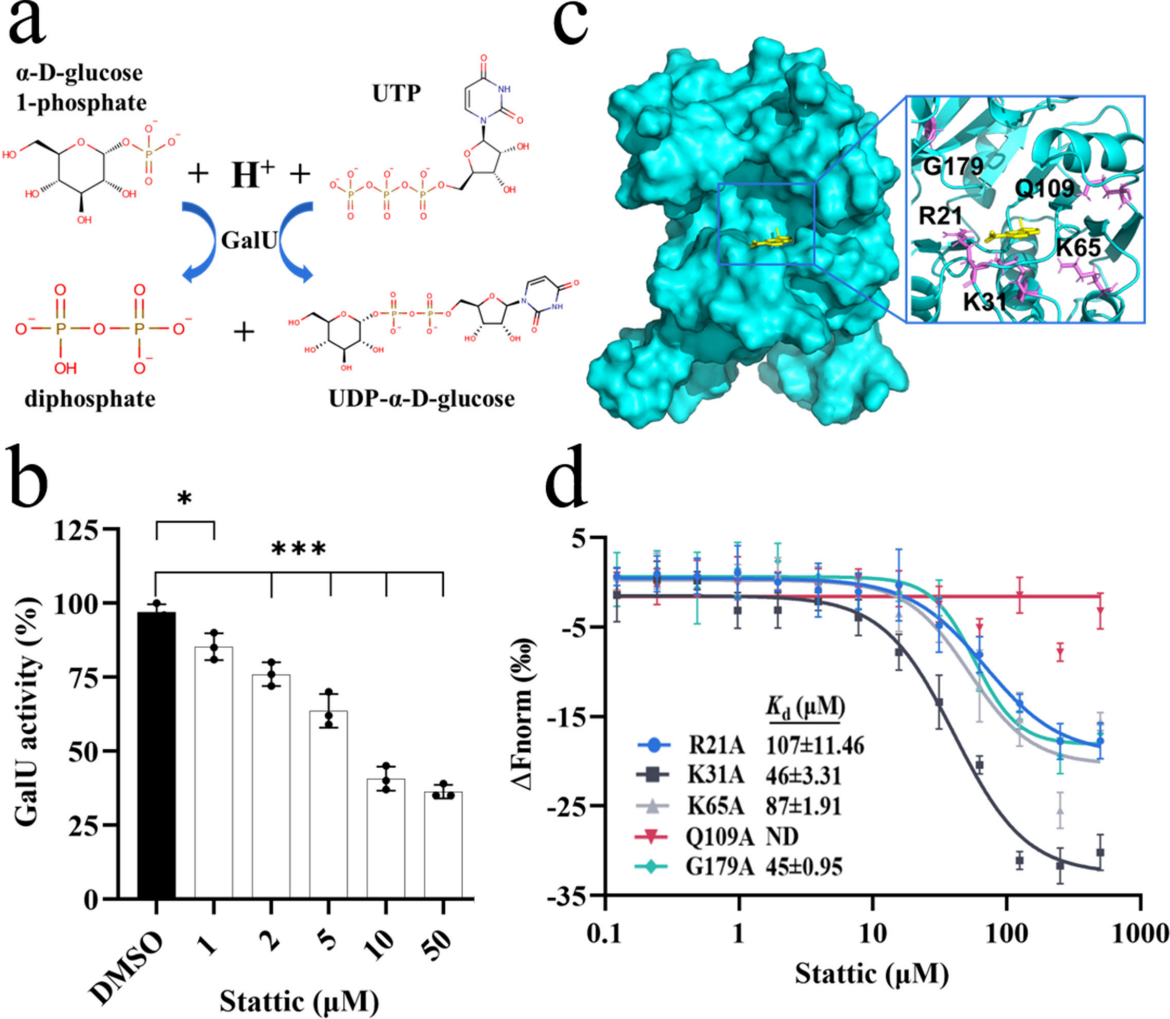
The effect of Stattic on the enzyme activity of GalU. (**a**) Catalytic reaction of GalU. (**b**) The change in GalU activity in the presence of different concentrations of Stattic. (**c**) The structure model of the binding of Stattic to GalU. Cyan represents the solid surface of GalU protein, yellow represents the stick model of Stattic, and magenta represents the amino acid residues that may interact with Stattic. (**d**) MST analysis of the binding of Stattic to GalU mutants. The data are presented as the mean ± SD (*n* = 3, independent measurements). One-way ANOVA was used to determine the significance of the results (*P < 0.05; **P < 0.01; ***P < 0.001; ns = no significance). ND, not detected.

GalU functions as a nucleotidyltransferase, catalyzing the formation of UDP-α-D-glucose and diphosphate (Fig. 3a). We tested whether the binding of Stattic to GalU affects the catalytic activity, and the malachite green reagent method was employed for detection (44). After incubating the protein for 10 min with various concentrations of Stattic, α-D-glucose 1-phosphate was added to initiate the reaction. The results demonstrated that the enzyme activity of GalU gradually decreased with increasing Stattic concentration and decreased by 60% when the concentration of Stattic was 50 µM (Fig. 3b). Then, we attempted to identify the Stattic-binding sites in *S. sonnei* GalU. The structure of *E. coli* homologous protein GalU was used as the docking template to predict the binding mode of Stattic and GalU by autodocking analysis. It was revealed that five amino acid residues, Arg21 (R21), Lys31 (K31), Lys65 (K65), Gln109 (Q109), and Gly179 (G179), might be critical for the interaction between GalU and Stattic. The docking model showed that the five residues are distributed near the active pocket of GalU, which may also contribute to the catalytic center (Fig. 3c). We then generated five single-point mutants (R21A, K31A, K65A, Q109A, and G179A). MST analysis showed that mutations at R21 and K65 markedly weakened the binding between GalU and Stattic, and the mutation of Q109 led to an absence of binding affinity between them (Fig. 3d). Moreover, GalU^Q109A^ enzyme activity was reduced by 35% compared to wild-type GalU (Table S2), and enzyme activity was unaffected when 10 µM Stattic was added, indicating that Q109 was not only the key site for binding but also for enzyme activity. In addition, R21 and G179 also affected the activity of GalU, which was consistent with previous findings (44, 45).

### Stattic inhibits pathogenic phenotypes and shows a good synergistic effect with antibiotics against *S. sonnei*

**Fig 4:**
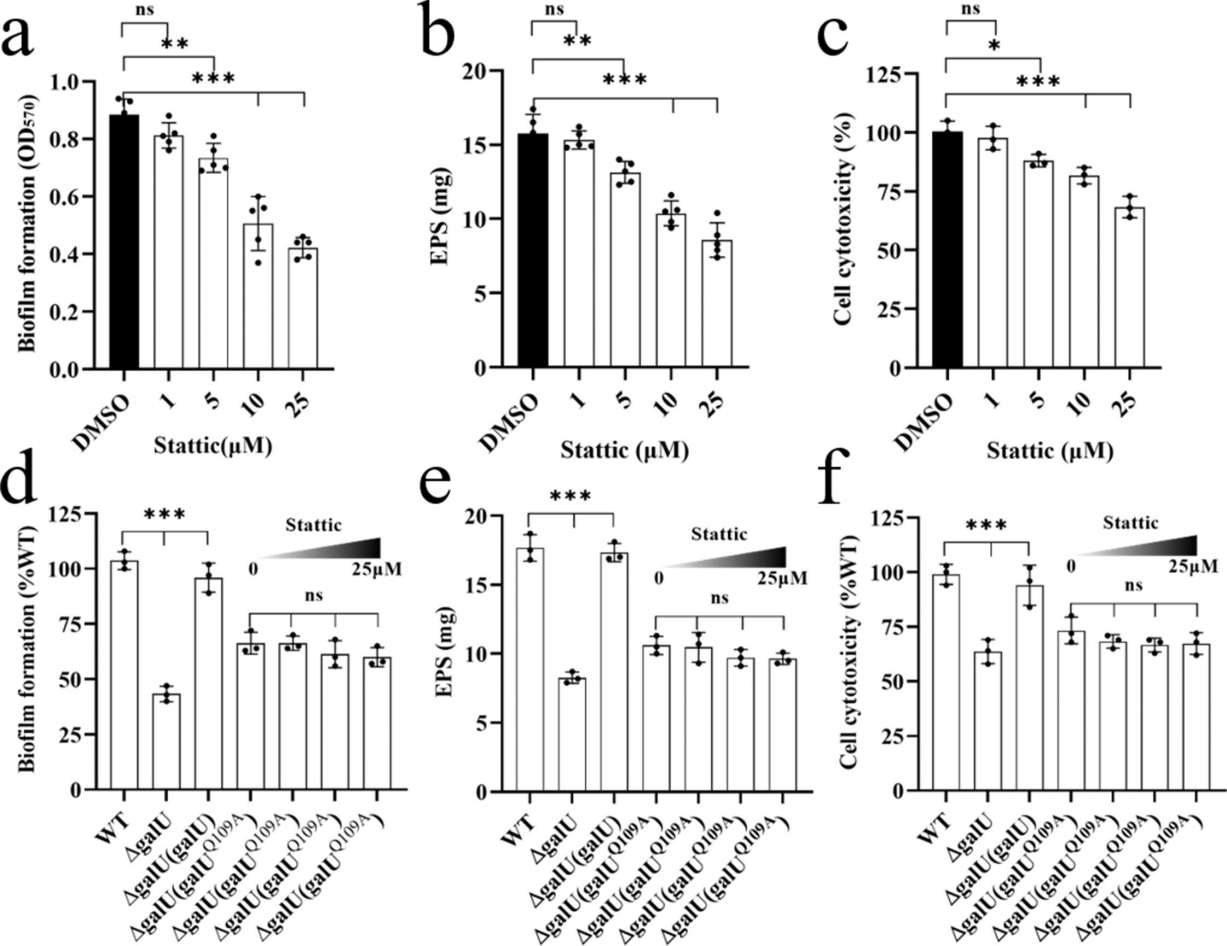
Effects of Stattic on the virulence-related phenotypes of *S. sonnei*. The virulence-related phenotypes of (**a**) biofilm formation (*n* = 5, independent measurements) and (**b**) EPS production (*n* = 5, independent measurements) in *S. sonnei* in the presence of different concentrations of Stattic were examined. (**c**) The cell cytotoxicity of *S. sonnei* in the presence of different concentrations of Stattic was evaluated by LDH assay (*n* = 3, independent measurements). The effects of Stattic on biofilm formation (**d**), EPS production (**e**), and cell cytotoxicity (**f**) of *S. sonnei* wild-type, *galU* mutant, *galU* complemented, and *galU* mutant complemented with *galU^Q109A^* were also examined (*n* = 3, independent measurements). *S. sonnei* was treated with different concentrations of Stattic and incubated statically at 37°C. The data are presented as the mean ± SD. Error bars indicate the SDs. One-way ANOVA was used to determine the significance of the results (*P < 0.05; **P < 0.01; ***P < 0.001; ns = no significance).

Stattic inhibited the activity of GalU in a dose-dependent manner (Fig. 3b). Thus, we then examined the effects of different concentrations from 1 to 25 µM of Stattic on the pathogenic phenotypes in *S. sonnei*, including biofilm formation, EPS production, and cell cytotoxicity. The results showed that biofilm formation, EPS production, and cell cytotoxicity were inhibited by Stattic in a dose-dependent manner, and exogenous addition of Stattic at 25 µM resulted in decreases in biofilm formation, EPS production, and cell cytotoxicity by 42%, 46%, and 32%, respectively, compared to those of the group without Stattic (Fig. 4a through c), and under this conditions, Stattic displayed slight toxicity to HeLa cells (Fig. S4). In addition, GalU^Q109A^ could only slightly restore the pathogenic phenotypes of *galU* deletion mutants, and the addition of Stattic did not inhibit the phenotypes of Δ*galU*(*galU^Q109A^*) (Fig. 4d through f), which suggested that Q109 of GalU is a crucial amino acid for Stattic binding and the biological functions of GalU in *S. sonnei*.

**Fig 5:**
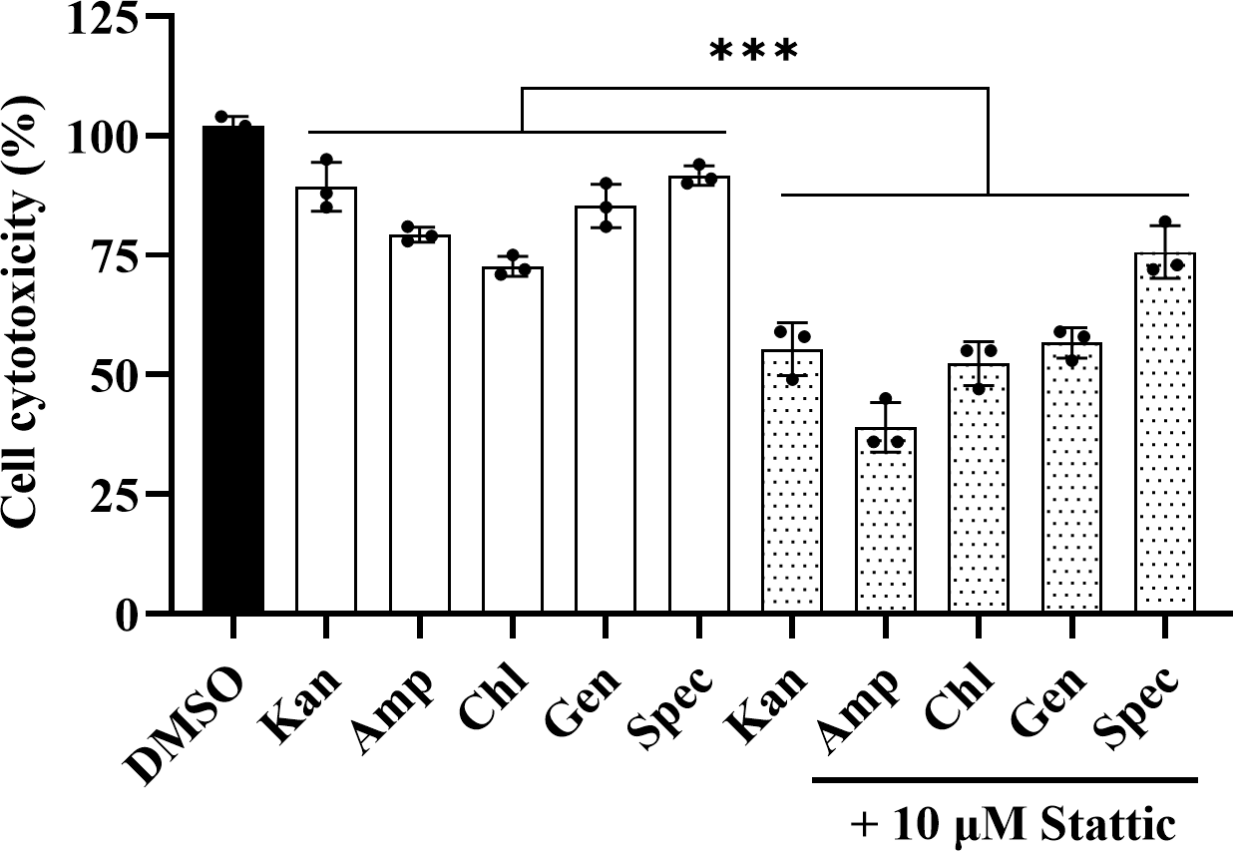
The synergistic effect of Stattic with different antibiotics on the cell cytotoxicity of *S. sonnei*. The following antibiotic concentrations were used in this experiment: Kan, kanamycin, 6.25 µg mL^−1^; Amp, ampicillin, 1.56 µg mL^−1^; Chl, chloramphenicol, 1.56 µg mL^−1^; Gen, gentamicin, 1.56 µg mL^−1^; and Spec, spectinomycin, 12.5 µg mL^−1^. The concentration of Stattic was 10 µM. The data are presented as the mean ± SD (*n* = 3, independent measurements). One-way ANOVA was used to determine the significance of the results (*P < 0.05; **P < 0.01; ***P < 0.001; ns = no significance).

Synergistic therapy is a common treatment strategy for severe and complex infections as well as for drug-resistant bacterial infections (46–48). We then tested the synergistic effect between Stattic and the commonly used antibiotics kanamycin, ampicillin, chloramphenicol, gentamicin, and spectinomycin on the treatment of *Shigella* infection (49). The results showed that when antibiotics were combined with Stattic, the inhibitory effect was enhanced, and the minimum inhibitory concentration (MIC) values were reduced to 6.25, 1.56, 1.56, 1.56, and 12.5 µg mL^−1^ from 12.5, 12.5, 3.125, 3.125, and 25 µg mL^−1^, respectively (Table 1), indicating that the Stattic and antibiotic combinations had a significant synergistic antibacterial effect. The determination of the total number of CFUs also suggested that Stattic had a good synergistic antibacterial effect (Fig. S5a and b). Furthermore, we continued to investigate whether Stattic has additive activity with antibiotics when used to treat HeLa cells infected by *S. sonnei*. The results showed that the combination of Stattic with antibiotics showed a stronger inhibitory effect on the virulence of *S. sonnei* than the antibiotics alone. Among them, the combination of ampicillin and Stattic exhibited a notable reduction of the virulence of *S. sonnei* by more than 65% (Fig. 5).

**TABLE 1:** Synergistic activities of Stattic at 10 µM with Kan, Amp, Chl, Gen, and Spec against the *S. sonnei* wild-type strain^*a*^

Compound (10 µM）	Kan MIC (µg mL^−1^)	Amp MIC (µg mL^−1^)	Chl MIC (µg mL^−1^)	Gen MIC (µg mL^−1^)	Spec MIC (µg mL^−1^)
None	12.5	12.5	3.125	3.125	25
Stattic	6.25	1.56	1.56	1.56	12.5

^*a*^ Kan, kanamycin; Amp, ampicillin; Chl, chloramphenicol; Gen, gentamicin; Spec, spectinomycin.

### Bactericidal activity of Stattic at high concentrations

Stattic did not affect the normal growth rate of *S. sonnei* cells when the concentration was less than 25 µM. However, we found that Stattic exhibited an obviously bactericidal activity when the concentration exceeded 100 µM (Fig. S6). The time-kill assay revealed that the number of bacterial cells decreased dramatically over time when *S. sonnei* was exposed to high concentrations of Stattic, and the deletion of *galU* did not affect the bactericidal efficacy of Stattic (Fig. S7a and b). Changes in the protein and nucleic acid content of bacterial culture medium supernatants can reflect bacterial damage. After 10 h, the protein and nucleic acid content in the supernatant of *S. sonnei* cells treated with Stattic increased significantly compared with the group without Stattic (Fig. S8a and b). Furthermore, when the bacterial cells were centrifuged and analyzed by SDS‒PAGE, the protein bands from *S. sonnei* cells treated with DMSO were significantly richer (Fig. S8c). The LIVE/DEAD *Bac*Light Bacterial Viability Kit, which includes the fluorescent dyes SYTO 9 and propidium iodide (PI), could also be used to assess the antibacterial properties of Stattic. The cells without Stattic treatment were stained with green fluorescence, and no red fluorescence was observed (Fig. S8d); however, when a high concentration of Stattic was added, the red fluorescence gradually increased (Fig. S8e and f), indicating that the bacterial cell membrane was damaged, which resulted in the nucleus being stained by PI. These results indicated that Stattic could destroy bacterial cell membranes, allowing bacterial contents to escape and causing irreversible damage. Furthermore, after exposure to Stattic, the bacterial cell membrane wrinkled, perforated, and even collapsed (Fig. S8g through i), and deletion of *galU* showed no effect on the demolishment of Stattic (Fig. S8j through o). Taken together, these results show that Stattic exerted good bactericidal activity at high concentrations.

### Development of drug-resistance of *S. sonnei* to Stattic

To reveal the bactericidal mechanism of Stattic, we tried to identify the target of Stattic by chemical mutagenesis. *S. sonnei* grew slowly in the presence of 50 µM Stattic (Fig. S5). After long-term induction, the growth rate of *S. sonnei* exposed to 50 µM Stattic increased significantly (Fig. S9a), indicating that it had developed resistance to Stattic. We then sequenced the genome of the mutant strain (named SY1) and compared it with that of the wild-type strain. There were three important variations in the mutant strain, including insertion or deletion (InDel), single nucleotide polymorphism (SNP), and copy number variation (CNV). The results of InDel and SNP showed that nine genes were mutated (Tables S3 and S4), and according to the base types before and after mutation, the mutant had no base preference, and the length of InDel was also within a reasonable range (Fig. S9b and c). The results of the functional analysis showed that four of them were transposases, and we tested whether the other five genes were associated with Stattic drug resistance. We overexpressed the five mutant genes in wild-type *S. sonnei*, which were named WT(*rpoS*), WT(*ipaH_1*), WT(*rhsA*), WT(*sson_1673*), and WT(*sson_0750*), respectively. Unfortunately, the results of the growth curve showed that the overexpression strains could not restore the growth rate to the *S. sonnei* SY1 strain when they were exposed to 50 µM Stattic (Fig. S9d). MST experiments also proved that the proteins encoded by these genes could not bind with Stattic (Fig. S10), indicating that these five proteins were not the key targets of Stattic. Therefore, we turned our attention to the last variant. A considerable increase or reduction in the copy number of large portions of the genome with a length of more than 1 kb is referred to as CNV, a complex phenomenon that is typically brought on by genomic rearrangement. The results of resequencing showed that there was a copy number increase in the gene segment found in the mutant SY1, and the gene length was 24,500 bp. By analyzing this gene fragment, we found that several genes encoding multiple antibiotic-resistance proteins were distributed in this region, mainly including MarR, MarA, MarB, and MarC (Fig. S9e). We overexpressed these genes in the wild-type strains, and the strains had increased resistance to Stattic (Fig. S9f). Therefore, we speculated that the resistance of the drug-resistant strain SY1 to Stattic is not caused by base mutations but by additional regulation of resistant proteins.

### Stattic inhibits the pathogenic phenotypes of *E. coli* and *K. pneumoniae*

**Fig 6:**
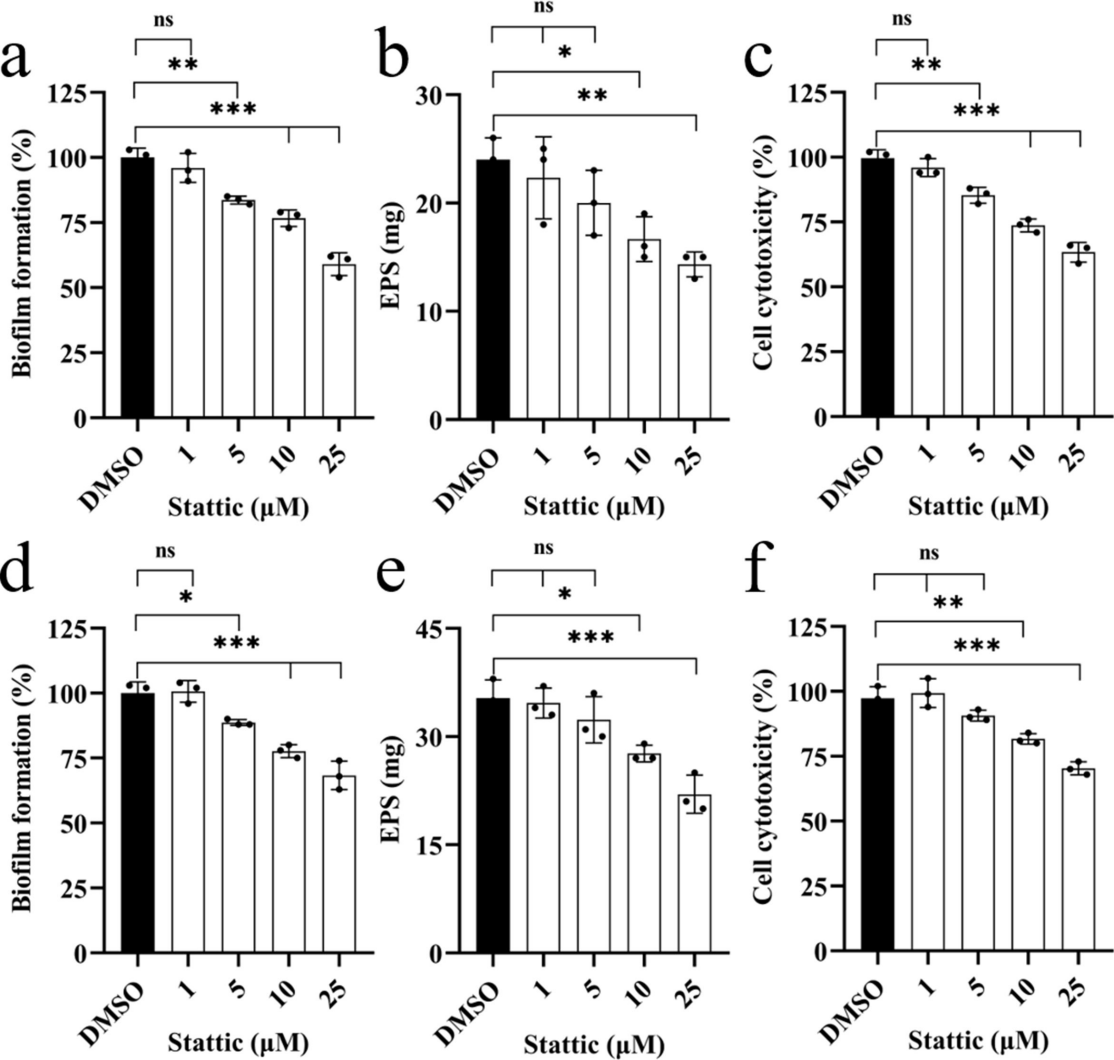
Effects of Stattic on the virulence-related phenotypes of *E. coli* and *K. pneumoniae*. The virulence-related phenotypes of biofilm formation (*n* = 3, independent measurements) (**a**), EPS production (*n* = 3, independent measurements) (**b**), and cell cytotoxicity (**c**) of *E. coli*, and those phenotypes (**d–f**) of *K. pneumoniae*, are presented with the addition of different concentrations of Stattic. The cell cytotoxicity of *E. coli* and *K. pneumoniae* in the presence of different concentrations of Stattic were evaluated by LDH assay (*n* = 3, independent measurements). The data are presented as the mean ± SD. Error bars indicate the SDs. One-way ANOVA was used to determine the significance of the results (*P < 0.05; **P < 0.01; ***P < 0.001; ns = no significance).

As the intestinal pathogens *E. coli* and *K. pneumoniae*, which belong to *Enterobacteriaceae*, have developed multiple drug resistance (50, 51), we then measured the effect of Stattic on *E. coli* and *K. pneumoniae* because the homologous protein of GalU in *S. sonnei* shares 100% and 94% identities with those in *E. coli* and *K. pneumoniae*. The results showed that Stattic inhibited the virulence-related phenotypes of *E. coli* and *K. pneumoniae* in a dose-dependent manner. Exogenous addition of 25 µM Stattic resulted in decreases in biofilm formation, EPS production, and cell cytotoxicity of *E. coli* by 41%, 40%, and 37% (Fig. 6a through c) and decreases in those of *K. pneumoniae* by 32%, 38%, and 30% (Fig. 6d through f). These results suggest that Stattic is a potential antimicrobial compound with a broad spectrum.

## DISCUSSION

*Shigella* is one of the main pathogens of intestinal infection that can cause shigellosis (6, 8). Shigellosis is a global human health problem and a major cause of diarrhea that causes approximately 700,000 deaths per year worldwide and millions of hospitalizations (52, 53). However, drug resistance in *S. sonnei* is a serious problem, so new antibiotics or therapies for disease treatment are needed (54, 55). The highly conserved protein GalU participates in the LPS core region biosynthetic process (22, 24), which is a critical virulence factor of bacteria. It has been reported that *galU* mutants in *Francisella tularensis* showed reduced virulence in a murine pulmonary model (56). In addition, Guo et al. showed that GalU affects the formation of biofilm *in planta of Xanthomonas citri* subsp., while mutation of *galU* resulted in a loss of pathogenicity in grapefruit (57). *Listeria monocytogenes* is a foodborne gram-positive pathogen, and studies have shown that the product encoded by *galU* is involved in wall teichoic acid galactosylation (58). The *galU* mutant was also severely reduced in a mouse oral-virulence model and shown to have defects in listeria actin-based motility; additionally, it even developed sensitivity to the antibiotic cefotaxime, which acts on the cell wall (35, 59). Previous studies have reported that the GalU protein functions as an EPS synthase involved in LPS biosynthesis in *Shigella* (22, 31). Our findings indicated that Stattic can bind to GalU with a *K*_d_ of 20 ± 0.35 µM, and we also discovered that the GalU homolog of *K. oxytoca* can also bind to Stattic with a *K*_d_ of 50 ± 0.25 µM, suggesting that GalU can not only be used as an effective antibacterial target for compounds to block the virulence of *S. sonnei* but also as a target for other bacterial species (Table S5).

Biofilm formation is an important pathogenic phenotype in *S. sonnei* and is one of the mechanisms of resistance used by bacteria. In addition, EPS is also an important virulence factor that promotes bacterial colonization and creates a pathological environment (60, 61). In this study, we discovered that Stattic has an excellent inhibitory effect on pathogenic phenotypes at low concentrations, which could inhibit its activity by directly binding to GalU and thus decreasing the yield of EPS as well as the formation of biofilm in a dose-dependent manner, ultimately reducing the virulence of *S. sonnei*. It was found that Q109 of GalU is the key binding site of Stattic, and mutation of it eliminated the inhibitory effect of Stattic. In addition, the residues of R21, K31, K65, and R179 also contribute to the activity of GalU. Our results demonstrated that Stattic has antimicrobial efficacy against *S. sonnei* by inhibiting GalU activity to interfere with the formation and secretion of virulence factors. This antibacterial mechanism is different from the direct germicidal efficacy of traditional antibiotics, which is beneficial to avoid the emergence of drug-resistant strains.

Controlling the use of antibiotics and reducing the pressure of antibiotic selection are the mainstream requirements to control the development of bacterial drug resistance. In gram-negative bacteria, *galU* was reported to be involved in the synthesis of polysaccharides, including LPS and CPS (22), which are important components used by bacteria to resist antibiotics (62, 63). Our results also showed that Stattic can be used in combination with many antibiotics to reduce their dosage (Table 1). One of the reasons is that Stattic can bind to GalU and inhibit LPS and CPS biosynthetic processes, thus increasing the sensitivity of *S. sonnei* to antibiotics. In addition, the combination of Stattic and antibiotics also reduced the toxicity of *S. sonnei* toward human cells (Fig. 5), suggesting that Stattic could potentially be developed as a new antimicrobial agent to treat *S. sonnei* infections and reduce the use of other antibiotics to prevent the development of drug resistance.

In addition, we found that Stattic also has germicidal effects at high concentrations. When *S. sonnei* is exposed to high concentrations of Stattic (>100 µM), its cell membrane will be destroyed, leading to content outflow, space collapse, and irreversible damage to *S. sonnei*. At the same time, we also discovered that *S. sonnei* can develop resistance to Stattic by upregulating the expression of multiple antibiotic-resistance genes, which proves that direct sterilization makes it easy to develop drug-resistant strains again. Stattic is a potent STAT3 inhibitor for tumor treatment (37, 38), but its antimicrobial activity has not yet been reported. Our results showed that Stattic has at least dual antibacterial mechanisms at different concentrations and a broad spectrum of antimicrobial activity, suggesting that it has better development prospects than traditional antibiotics.

## MATERIALS AND METHODS

### Strains, culture, and agents

Table 2 contains a list of the bacterial strains employed in this study. The plasmids and bacterial strains used in this study were all sequenced. Unless otherwise specified, *S. sonnei* and *E. coli* strains were cultured in Luria-Bertani (LB) medium (5 g/L yeast extract, 10 g/L tryptone, 10 g/L NaCl; pH 7.4) or LB medium with 15 g/L agar at 37°C. Antibiotics were added to the medium based on the needs of the experiment, and the following antibiotics were employed in this work: ampicillin (100 µg/mL), chloramphenicol (50 µg/mL), kanamycin (100 µg/mL), gentamicin (50 µg/mL), or spectinomycin (100 µg/mL). Stattic (CAS 19983–44-9) was dissolved in DMSO before dilution, which was purchased from Solarbio.

**TABLE 2:** Bacterial strains and plasmids used in this study

Strain or plasmid	Phenotype and/or characteristic(s)^*a*^	Source or reference
*S. sonnei*		
CMCC51592	Wild-type strain of *S. sonnei*	Laboratory collection
*S. sonnei(pMar*)	*S. sonnei* harboring the expression construct *pMar*	This study
Δ*galU*	*S. sonnei* with *galU* being deleted	This study
Δ*galU*(*galU*)	Mutant Δ*galU* harboring the expression construct pUC*-galU*	This study
Δ*galU(galU*^Q109A^)	Mutant Δ*galU* harboring the expression construct pUC*-galU^Q109A^*	This study
SY1	*S. sonnei* mutant strain induced by Stattic	This study
WT(*rpoS*)	*S. sonnei* harboring the expression construct pUC*-rpoS*	This study
WT(*ipaH_1*)	*S. sonnei* harboring the expression construct pUC*-ipaH_1*	This study
WT(*rhsA*)	*S. sonnei* harboring the expression construct pUC*-rhsA*	This study
WT(*sson_1673*)	*S. sonnei* harboring the expression construct pUC*-sson_1673*	This study
WT(*sson_0750*)	*S. sonnei* harboring the expression construct pUC*-sson_0750*	This study
*E. coli*		
DH5α	*supE44 lacU169*(80*lacZ* M15) *hsdR17 recA1 endA1 gyrA96 thi-1 relA1 pir*	Laboratory collection
BL21	F^−^ *ompT hsdS* (r_B_^−^m_B_^−^) *dcm1^+^* + gal (DE3) *endA*	Laboratory collection
ATCC35150	Serotype O157:H7	Laboratory collection
*K. quasipneumoniae*		
ATCC700603	Extended-spectrum beta-lactamase production	Laboratory collection
Plasmids		
pET28(a)+	Expression vector, Kan^r^	Laboratory collection
pET-*galU*	pET28a containing *galU*	This study
pET-*galU^R21A^*	pET28a containing *galU^R21A^*	This study
pET-*galU^K31A^*	pET28a containing *galU^K31A^*	This study
pET-*galU^K65A^*	pET28a containing *galU^K65A^*	This study
pET-*galU^Q109A^*	pET28a containing *galU^Q109A^*	This study
pET-*galU^G179A^*	pET28a containing *galU^G179A^*	This study
pET-*rpoS*	pET28a containing mutant *rpoS*	This study
pET-*ipaH_1*	pET28a containing mutant *ipaH_1*	This study
pET-*rhsA*	pET28a containing mutant *rhsA*	This study
pET-*sson_0750*	pET28a containing mutant *sson_0750*	This study
pET-*sson_1673*	pET28a containing mutant *sson_1673*	This study
pET-*galU_K.o_*	pET28a containing *galU_K.o_*	This study
pET-*galU_A.b_*	pET28a containing *galU_A.b_*	This study
pET-*galU_B.c_*	pET28a containing *galU_B.c_*	This study
pET-*galU_P.a_*	pET28a containing *galU_P.a_*	This study
pUC18	Broad-host-Range cloning vector, Amp^r^	Laboratory collection
pUC*-rpoS*	pUC18 containing mutant *rpoS*	This study
pUC*-ipaH_1*	pUC18 containing mutant *ipaH_1*	This study
pUC*-rhsA*	pUC18 containing mutant *rhsA*	This study
pUC*-sson_1673*	pUC18 containing mutant *sson_1673*	This study
pUC*-sson_0750*	pUC18 containing mutant *sson_0750*	This study
p*Mar*	pUC18 containing *marA*, *marB*, *marC*, and *marR*	This study
pKD3	Template for amplifying the *cat* gene	Laboratory collection
pKD46	λ Red recombinase expression, Amp^r^	Laboratory collection

^*a*^ Kan^r^, resistance to kanamycin; Amp^r^, resistance to ampicillin.

### Autodocking

The target GalU structure (PDB: 2E3D) was obtained from the Protein Data Bank (https://www.rcsb.org/), and the protein contains four chains, A, B, C and D, which form a homotetramer. Chain A was used for the dock model. AutoDock 4.2.1 was used for all dockings in this study, which was supported by Autodock tools and MGL tools. It was also used for protein optimization, including deleting water, adding the polar hydrogen group, and Computing Gasteiger. Torsion bonds of the compounds were selected and defined. The docking parameters were kept to their default values. The receptor grid was centered at *x*  =  −9.176, *y*  =  42.570, and *z*  =  13.969, and the grid spacing changed from 0.375 to 0.44. Ligand tethering of the protein was accomplished by adjusting the genetic algorithm (GA) parameters throughout the course of 10 iterations. The dock results of complex conformation ranked by energy were displayed in PyMOL, which is the most popular protein visualization software.

### Protein expression and purification

After cloning the intact *galU* gene into the pET28a (+) vector, it was transformed into competent *E. coli* BL21 (DE3) cells. Then, the LB medium with 100 g/mL kanamycin was used for protein expression. Up to an OD_600_ of 0.4–0.6, cells were grown at 37°C and 220 rpm. 1 mM isopropyl-D-1-thiogalactopyranoside was added to induce recombinant gene expression overnight at 16°C. Cells were collected by centrifugation at 4,000 × *g* for 30 min after induction. Cells were disturbed by sonication after being resuspended in phosphate buffered saline (PBS) (pH 7.4). A 1 mL His-Tag column (GE Healthcare) that had previously been equilibrated with PBS was loaded with the supernatant after the resulting suspension had been centrifuged for 30 min at 4°C and 12,000 g. The PBS containing 250 mM NaCl were used to wash the column. Finally, the target proteins were eluted with 250 mM imidazole in PBS buffer, which was placed into gels (GenScript SurePAGE), and the purity was evaluated by SDS‒PAGE.

### Enzyme activity assays

The colorimetric method described previously was used to determine GalU activity at 37°C (44, 64). In brief, the GalU in a suitable dilution was put into the reaction mixtures (MgCl_2_,10 mM; yeast inorganic pyrophosphatase, 0.5 U/mL; Uridine 5'-triphosphate (UTP), 1 mM; bovine serum albumin (BSA), 0.5 mg/mL; 4-propanesulfonyl morpholine (MOPS), pH 8.0, 50 mM). 2 mM glucose-1-phosphate and Malachite Green were added to the reaction mixtures as initiator and terminator, respectively. The GalU activity was measured using a microplate reader set to 630 nm.

### MST assays

The binding affinity of compounds and GalU was detected by using the MST. Briefly, the purified GalU was labeled using the Protein Labeling Kit. Fluorescence was measured after labeled protein, and ligand concentrations were loaded onto silicon capillaries that had undergone standard treatment (MO-K025, NanoTemper Technologies). The binding affinity was measured and carried out at automatic LED power and 40% MST power on a Monolith NT.115 instrument.

### Construction of the *S. sonnei* mutant and complemented strains

The *galU* mutants were generated by an in-frame deletion strategy using the λ Red recombinase system as described previously (40). In brief, the PCR product, which contains the 39 bp homologous arm sequences and the chloramphenicol-resistant gene amplified from pKD3, was transferred into the competent *S. sonnei* containing pKD46. Besides, the pUC18 was used to construct the complemented plasmid. The mutants and complemented strains were identified by PCR and Sanger sequencing, and the primers used in this study were all listed in Table 3.

**TABLE 3:** PCR primers used in this study^*a*^

Primer	Sequence (5′−3′)
For deletion	
*galU*-KO-F	TGAACACGTTCAAAACACGAACAGTCCAGGAGAATTTAAGTGTAGGCTGGAGCTGCTTC
*galU*-KO-R	TTGCTCAACGCCGTTTCGTGGATAACACCGATACGGATGATGGGAATTAGCCATGGTCC
For deletion confirmation	
*galU*-out-F	CTAACTCGCCTCCTTTTCAGA
*galU*-out-R	CCGGTTTAAGACAATTTAATAAG
For recombinant protein expression	
*galU*-F	CGGGATCCATGGCTGCCATTAATACGAAA
*galU*-R	CGGAATTCTTACTTCTTAATGCCCATCTC
*galU_K.o_*-F	CGGGATCCATGGCTGCGCTTAATTCA
*galU_K.o_*-R	CGGAATTCTTACTTCGCTACGGCTTTAT
*galU_A.b_*-F	CGGGATCCATGATTAAAAAAGCAGTTTTA
*galU_A.b_*-R	CGGAATTCCTATAATTTAAGTTCCTGAATT
*galU_B.c_*-F	CGGGATCCATGTTGAAAGTCACCAAGGC
*galU_B.c_*-R	CCCAAGCTTTCAGACGGTCGGTTGAGC
*galU_P.a_*-F	CGGGATCCATGATCAAGAAATGTCTTTTCC
*galU_P.a_*-R	CGGAATTCTCAGTGAGCCTTGCCGGT
*rpoS*-F	CGGGATCCATGAGTCAGAATACGCTGAA
*rpoS*-R	CGGAATTCTTATTGTGCACAGAAAAGGC
*ipaH_1*-F	CGGGATCCATGAAACCTGCCCACAATC
*ipaH_1*-R	CGGAATTCTTATGAATGGTGCAGTTGTG
*sson_0750-*F	CGGGATCCATGCACATGTCTCTGGCG
*sson_0750-*R	CGGAATTCTTACTCAACGTCAAACGCCC
*rhsA*-F	CAGCAAATGGGTCGCGGATCCATGCGAATCGCTGATCGAA
*rhsA*-R	TTGTCGACGGAGCTCGAATTCTCACCGTAATTGAGTTGTTG
*sson_1673-*F	CAGCAAATGGGTCGCGGATCCATGAGCGGAAAACCGGCGG
*sson_1673-*R	TTGTCGACGGAGCTCGAATTCTCAGCACCTGCCCGGTCTG
For point mutation	
*galU*-R21A-F	GTTGCGGGATTAGGAACCGCGATGTTGCCGGCGACGAA
*galU*-R21A-R	TTCGTCGCCGGCAACATCGCGGTTCCTAATCCCGCAAC
*galU*-K31A-F	CGACGAAAGCCATCCCGGCAGAGATGCTGCCACTTG
*galU*-K31A-R	CAAGTGGCAGCATCTCTGCCGGGATGGCTTTCGTCG
*galU*-K65A-F	CTGGTTACACACTCATCTGCAAACTCTATTGAAAACCAC
*galU*-K65A-R	GTGGTTTTCAATAGAGTTTGCAGATGAGTGTGTAACCAG
*galU*-Q109A-F	ACTATTATGCAAGTTCGTGCGGGTCTGGCGAAAGGCCT
*galU*-Q109A-R	AGGCCTTTCGCCAGACCCGCACGAACTTGCATAATAGT
*galU*-G179A-F	GCTGATGTGACCGCATATGCCGTTGTGGATTGCAAAGGC
*galU*-G179A-R	GCCTTTGCAATCCACAACGGCATATGCGGTCACATCAGC
For *in trans* expression	
*galU-*pUC-F	CGGAATTCATGGCTGCCATTAATACGAAA
*galU-*pUC-R	CGGGATCCTTACTTCTTAATGCCCATCTC
*galU*(Q109A)*-*pUC-F	CGGAATTCATGGCTGCCATTAATACGAAA
*galU*(Q109A)*-*pUC-R	CGGGATCCTTACTTCTTAATGCCCATCTC
*pMar*-F	CGGAATTCTTAATGGTACGTTTTAATGATTTC
*pMar*-R	CGGGATCCCTACATAGCGTGTTGATTATAATAG
*rpoS-*pUC-F	CGGAATTCATGAGTCAGAATACGCTGAA
*rpoS-*pUC-R	CGGGATCCTTATTGTGCACAGAAAAGGC
*ipaH_1-*pUC-F	CGGAATTCATGAAACCTGCCCACAATC
*ipaH_1-*pUC-R	CGGGATCCTTATGAATGGTGCAGTTGTG
*sson_0750-*pUC-F	CGGAATTCATGCACATGTCTCTGGCG
*sson_0750-*pUC-R	CGGGATCCTTACTCAACGTCAAACGCCC
*rhsA-*pUC-F	TATGACCATGATTACGAATTCATGCGAATCGCTGATCGAAC
*rhsA-*pUC-R	CAGGTCGACTCTAGAGGATCCTCACCGTAATTGAGTTGTTGACCA
*sson_1673-*pUC-F	TATGACCATGATTACGAATTCATGAGCGGAAAACCGGCGG
*sson_1673-*pUC-R	CAGGTCGACTCTAGAGGATCCTCAGCACCTGCCCGGTCTG

^*a*^ Restriction enzyme sites are underlined. F, forward; R, reverse.

### Biofilm formation assays

The biofilm formation assays were carried out by using crystal violet staining as previously described (40). In brief, the OD_600_ of fresh *S. sonnei* was diluted to 0.05 and added to each well of a 96-well polystyrene plate in the absence or presence of different concentrations of Stattic, and the samples without shaking were incubated at 37°C for 12 h. Then, remove the supernatant carefully and gently rinse with PBS three times. The samples were fixed with methanol and dried in an oven at 45°C, 150 µL of 0.5% crystal violet was added for staining and then washed with PBS to fully remove the crystal violet. Finally, the samples were dissolved by adding 150 µL of 95% ethanol. The OD_570_ was measured using the Multiskan Spectrum.

### Quantification of EPS

The same procedures as reported above were used to prepare the *S. sonnei* suspensions (OD_600_ = 0.05). The samples with or without different concentrations of Stattic were cultured at OD_600_ = 3.0 at 37°C with shaking, centrifuged at 12,000 × *g* for 30 min at 4°C, the supernatant was collected and incubated with two times the volume of absolute ethanol for 12 h at 4°C and then centrifuged at 12,000 × *g* for 10 min again. After removing the supernatant, the precipitate was air-dried, and the weight was calculated by using a one-hundred-thousandth analytical balance.

### Cell cytotoxicity assays

Cytotoxicity assays were carried out in accordance with previously described procedures (40). In brief, HeLa cells (1 × 10^5^ cells/well) were co-incubated with fresh *S. sonnei* cells at 10^9^ colony-forming units (CFU)/mL for 8 h in dulbecco's modified eagle medium (DMEM) (1% fetal bovine serum). The CytoTox 96 Kit (G1780, Promega) was used to measure the content of LDH released from infected cells. The cytotoxicity experiment results were assessed by detecting the OD_490_ and calculating the cytotoxicity compared to an uninfected control.

### Growth rate analysis

The same procedures as reported above were used to prepare the *S. sonnei* suspensions (OD_600_ = 0.05). The samples were incubated at 37°C with shaking, and the OD_600_ was detected every 4 h. Each experiment was repeated three times.

### Time-kill assay

The time-kill assay was carried out as described previously (65). Antibiotics with or without Stattic were added to *S. sonnei* suspensions (7 log CFU/mL). LB broth containing DMSO was used as the control group. The samples were incubated at 37°C, and the medium was taken at 0, 4, 8, 12, 18, and 24 h, which was diluted with sterile water and spread on a non-resistant LB plate overnight. Finally, the number of bacteria was calculated.

### Determination of MICs

The MICs of antibiotics were estimated using the microdilution method described previously (65). In brief, antibiotics were added to 96-well plates with 150 µL of *S. sonnei* suspensions (OD_600_ = 0.05) at seven different final concentrations (50 µg/mL, 25 µg/mL, 12.5 µg/mL, 6.25 µg/mL, 3.125 µg/mL, 1.56 µg/mL, and 0.78 µg/mL). The MIC was determined by measuring the OD_600_ nm on a multimode microplate reader. For the combination therapy assay, Stattic was added to the mixture at a final concentration of 10 µM. The plates were then incubated at 37°C for 24 h without shaking, and the follow-up experimental steps were consistent with the time-kill assay.

### Determination of nucleic acid/protein leakage

The leakage of nucleic acids and proteins through the *S. sonnei* cell membrane was measured according to the method described previously (65). Briefly, *S. sonnei* cells suspended in sterile water were treated with different concentrations of Stattic (0, 100 µM, and 200 µM) at 37°C for 10 h. By centrifuging at 12,000 × *g* for 15 min at 4°C, the extracellular nucleic acid and protein content of the supernatants were determined using a NanoPhotometer (Implen N60 Touch). Intracellular protein analysis was performed by SDS-PAGE.

### Determination of membrane integrity by confocal laser scanning microscopy

Bacterial suspensions (OD_600_ = 0.5) treated with different concentrations of Stattic were incubated at 37°C overnight. Before staining, samples were washed twice with PBS. Then, fluorescent dyes (SYTO 9 and PI) were added, and the mixtures were incubated in the dark for 10 min. Samples were washed twice with PBS and finally resuspended in PBS. Eightmicroliters of the suspension stained with SYTO 9 and PI were dropped on a microscope glass slide and observed using a confocal laser scanning microscopy (CLSM).

### Determination of morphological changes by scanning electron microscopy

Bacterial suspensions (OD_600_ = 0.5) treated with different concentrations of Stattic (0, 100 µM, and 200 µM) were incubated at 37°C for 4 h. After incubation, the samples were centrifuged (4°C, 4,000 × *g*, 5 min) and washed twice with PBS. Then, 2.5% glutaraldehyde in PBS was added to resuspend the cell pellets, and the resulting samples were incubated at 4°C for 10 h. The cell pellets were collected and centrifuged at 4,000 × *g* for 5 min at 4°C and then washed twice with PBS. The 30%, 50%, 70%, 80%, 90%, and 100% water-ethanol solutions were used for the gradient dehydration of the samples. Finally, samples were treated with critical point drying and gold spray and observed using a SEM.

### Screening of the Stattic-resistant *S. sonnei* mutant strain and whole-genome resequencing

The method of successive generations was used to develop the resistance of *S. sonnei* to Stattic. In brief, Stattic at a final concentration of 50 µM was added to the *S. sonnei* suspensions (OD_600_ = 0.05) and then incubated for 24 h at 37°C. Fresh *S. sonnei* suspensions with or without 50 µM Stattic were used as negative or positive control groups, respectively. Successive generations were performed until there was a significant difference in growth rates between the experimental group and the negative control group. Finally, whole-genome resequencing of the Stattic-resistant *S. sonnei* mutant strain and *S. sonnei* wild-type strain was carried out by Shanghai Personalbio Biotechnology (China) using the Illumina HiSeq sequencing platform (paired-end, 2  ×  150 bp). The genome sequences are deposited in the National Center for Biotechnology Information (NCBI) BioProject repository under the accession number PRJNA1025100, and the BioSample numbers of *S. sonnei* wild-type strain and mutant SY1 strain are SAMN37717590 and SAMN37724331, respectively.

### Statistical analysis

Statistical analyses were performed using GraphPad Prism 8. All experiments were performed at least three times independently. Data are presented as the mean ± SD. Unpaired *t* tests between two groups, one-way analysis of variance, or two-way analysis among multiple groups were used to calculate *P* values. *P* values are reported using the following symbolic representations: ns (no significance), *P < 0.05, **P < 0.01, and ***P < 0.001.

## Data Availability

Data supporting the findings of this study are available within the paper and its supplementary information files.

## SUPPLEMENTAL MATERIAL

Supplemental figures and tables aem.01074-23-s0001.docx Fig. S1-S10, Table S1-S5

ASM does not own the copyrights to Supplemental Material that may be linked to, or accessed through, an article. The authors have granted ASM a non-exclusive, world-wide license to publish the Supplemental Material files. Please contact the corresponding author directly for reuse.
