# Supplementary material for: Anti-virulence and bactericidal activities of Stattic against Shigella sonnei
Source: Appl Environ Microbiol. 2023 Nov 30;89(12):e01074-23. doi: 10.1128/aem.01074-23 (PMC10734500; doi:10.1128/aem.01074-23)
Supplement: Supplemental figures and tables — Fig. S1-S10, Table S1-S5 [file aem.01074-23-s0001.docx]

**Anti-virulence and bactericidal activities of Stattic against *Shigella sonnei***

Mingfang Wang ^a, #^, Jia Zeng ^a, #^, Huihui Tan ^a^, Quan Guo ^a^, Xia Li ^a^, Xiwen Ling ^a^, Jinyue Zhang ^b^, Shihao Song ^a,b^, Yinyue Deng ^a,^*

^a^ *School of Pharmaceutical Sciences (Shenzhen), Shenzhen Campus of Sun Yat-sen University, Sun Yat-sen University, Shenzhen 518107, China*

^b^ *School of Pharmaceutical Sciences, Hainan University, Haikou 570228, China*

^#^These authors contributed equally to this work.

***To whom correspondence may be addressed:**

Yinyue Deng, Email: dengyle@mail.sysu.edu.cn

**Running title: Antimicrobial activities of Stattic against *S. sonnei***


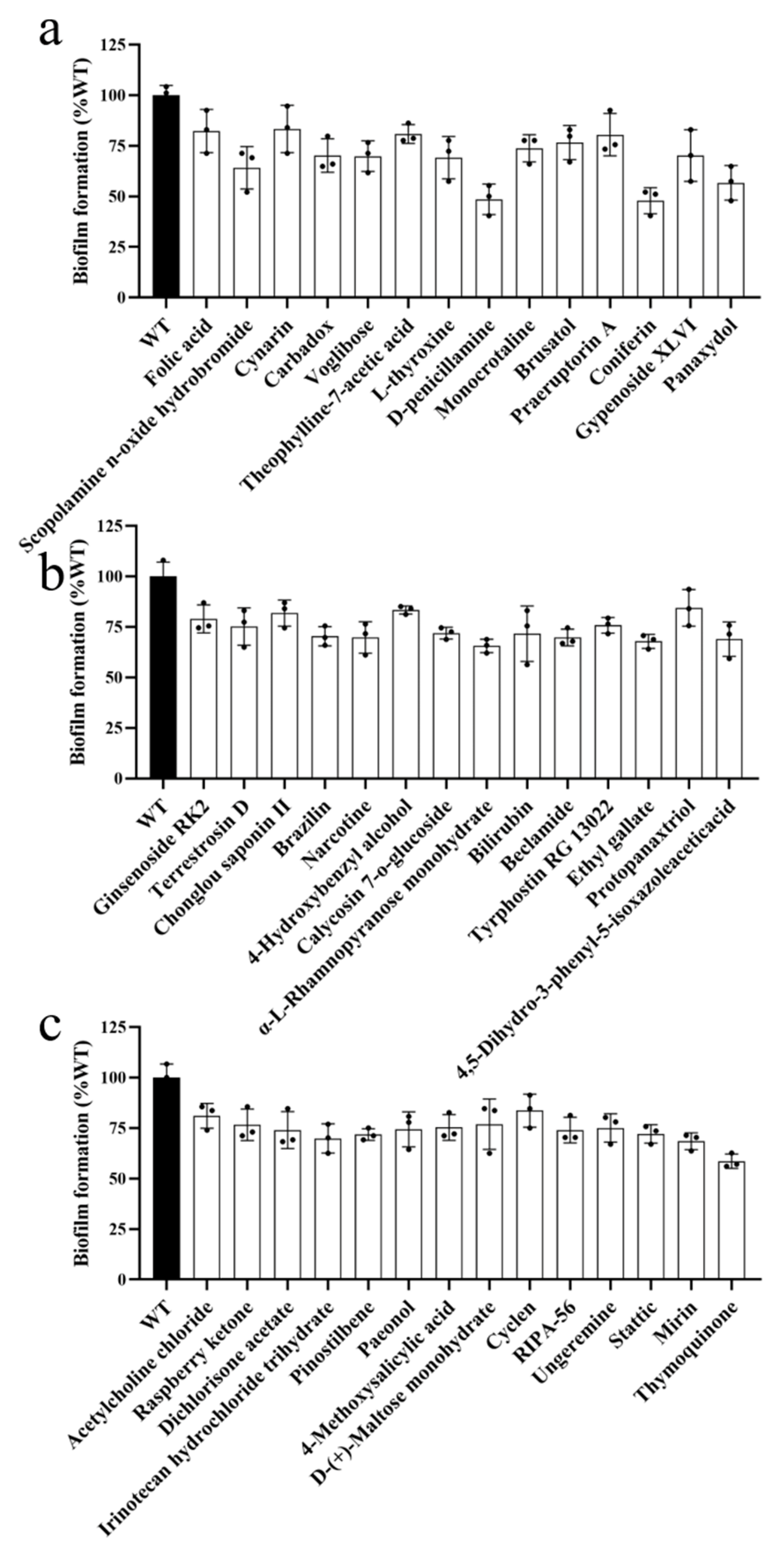


**Fig. s1** Biofilm formation of *S. sonnei* in the presence of different compounds at a final concentration of 20 μM. Compounds were dissolved in DMSO, and the same volume of DMSO used as the solvent for the compounds was used as a control. The data are presented as the mean ± SD (n = 3, independent measurements).


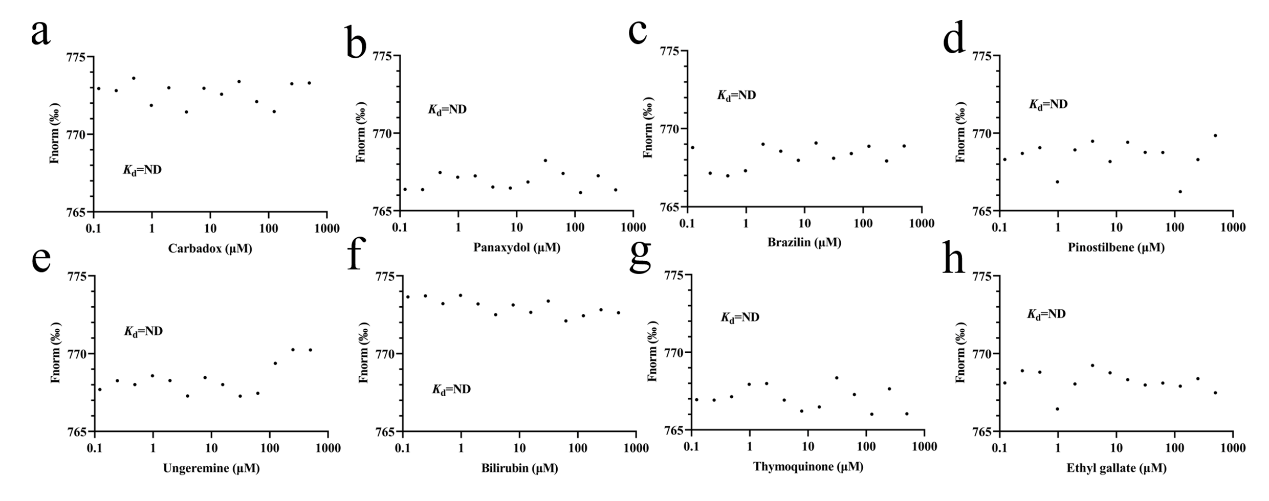


**Fig. s2** MST analysis of the binding of GalU to different candidate compounds.


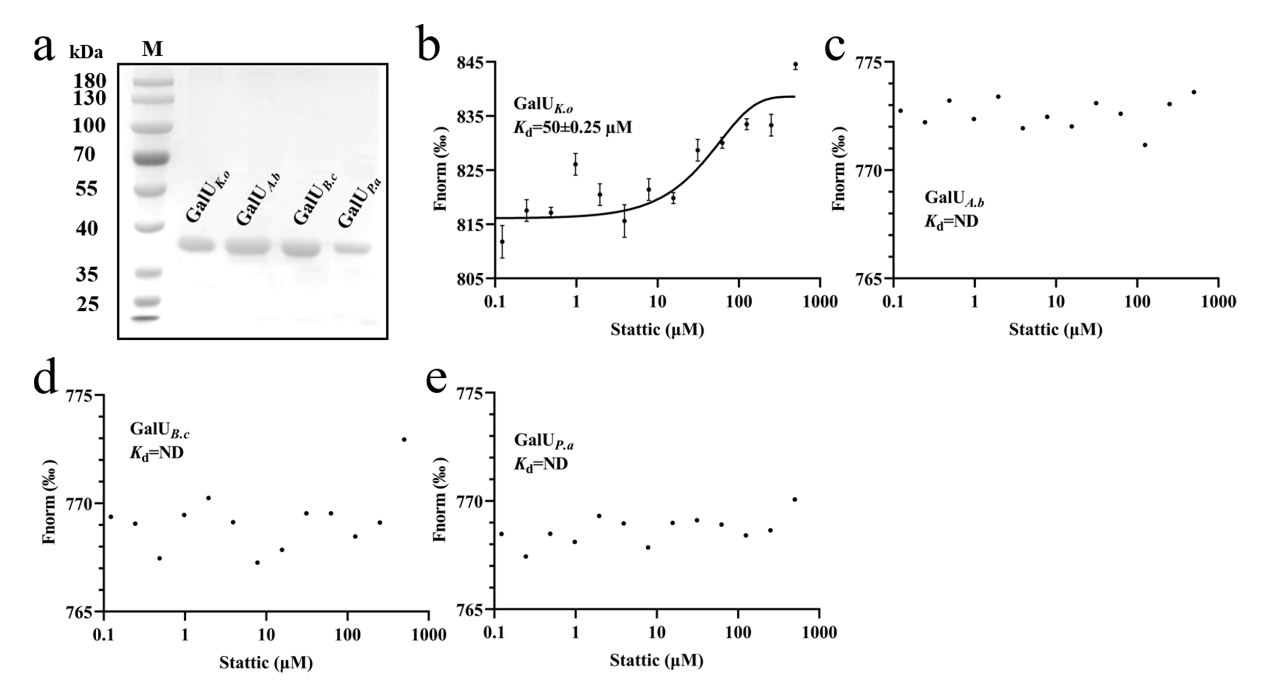


**Fig. s3** MST analysis of the binding of Stattic to GalU homologous protein. (a) SDS‒PAGE analysis of the four GalU homologous proteins. (b-e) The detection of the binding of four GalU homologous proteins to Stattic by using MST. ND, not detected.


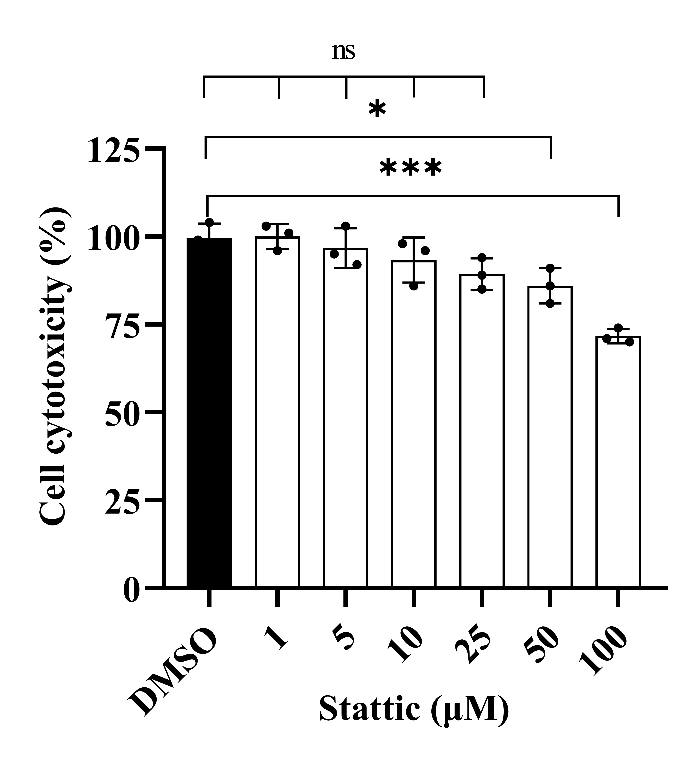


**Fig. s4** Toxicity of different concentrations of Stattic on HeLa cells. The data are presented as the mean ± SD. Error bars indicate the SDs. One-way ANOVA was used to determine the significance of the results (**p* <0.05; ***p* < 0.01; ****p* < 0.001; ns = no significance).


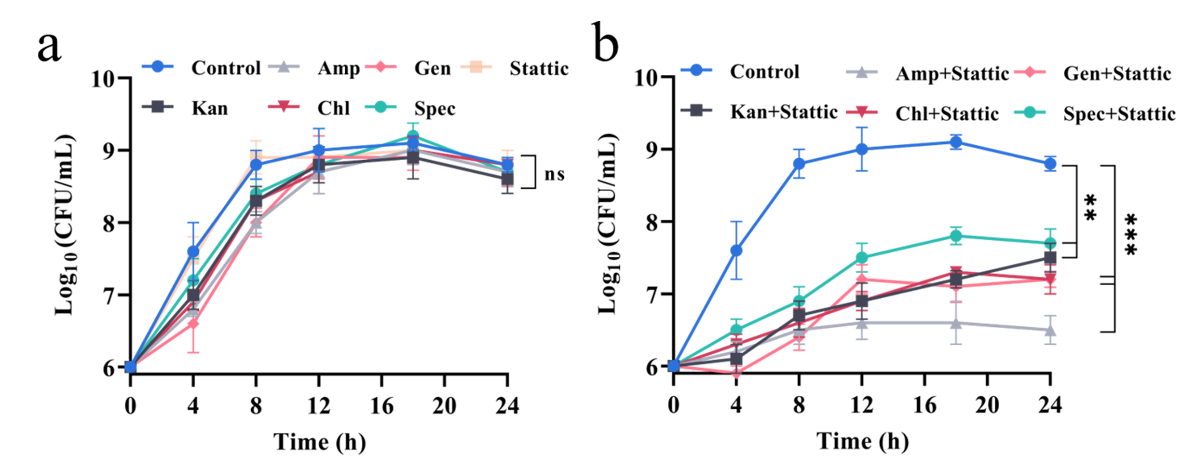


**Fig. s5** Combination therapy efficacy of Stattic combined with antibiotics. The change in the number of bacterial cells over time in the presence of antibiotics without (a) or with Stattic (b). The following antibiotic concentrations were used in this experiment: Kan, kanamycin, 6.25 µg mL^-1^; Amp, ampicillin, 1.56 µg mL^-1^; Chl, chloramphenicol, 1.56 µg mL^-1^; Gen, gentamicin, 1.56 µg mL^-1^; and Spec, spectinomycin, 12.5 µg mL^-1^. The concentration of Stattic was 10 µM. The initial number of *S. sonnei* in all samples was 6 log CFU/mL, and the data are presented as the mean ± SD (n = 3, independent measurements). One-way ANOVA was used to determine the significance of the results (Time = 24 h, **p* <0.05; ***p* < 0.01; ****p* < 0.001; ns = no significance).


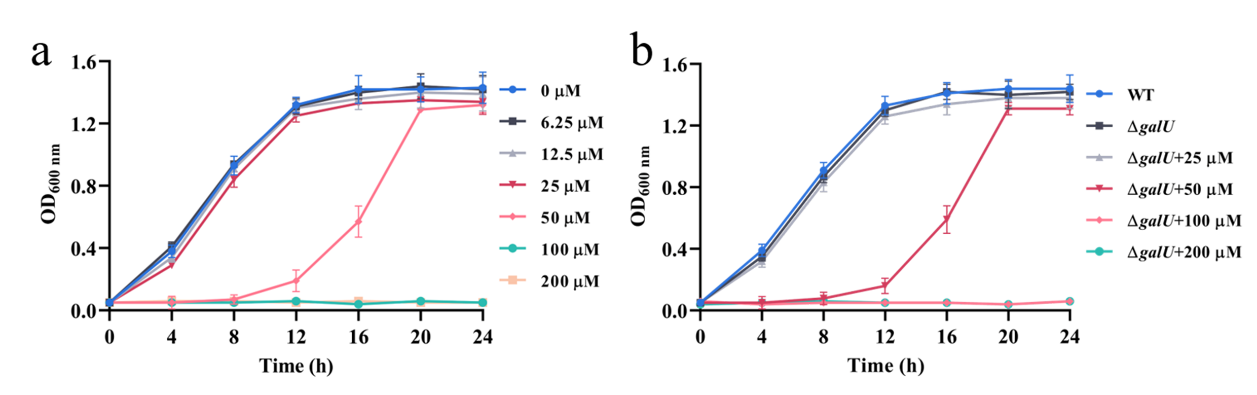


**Fig. s6** Growth curve of *S. sonnei* WT strains (a) and *galU* mutant strains (b) treated with different concentrations of Stattic (0 to 200 μM). The data are presented as the mean ± SD (n = 3, independent measurements).


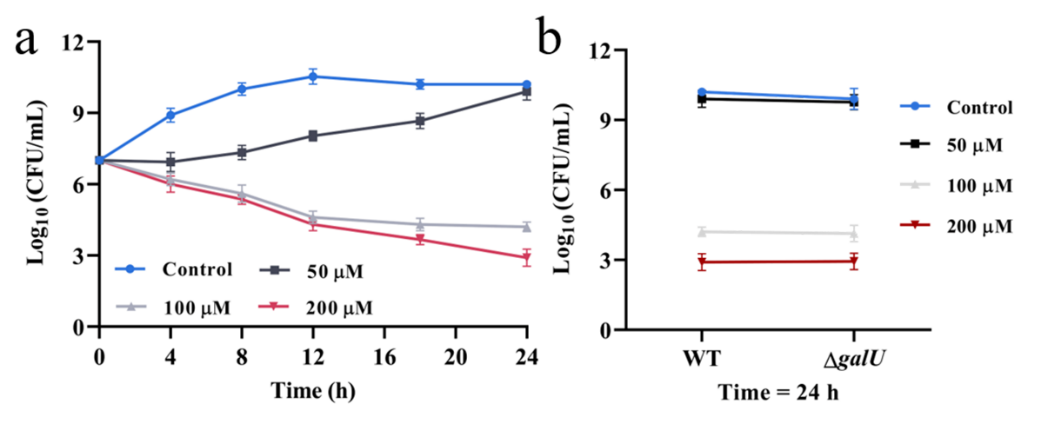


**Fig. s7** Time-kill curves of *S. sonnei* WT strains (a) and *galU* mutant strains (b) in the presence of different concentrations of Stattic. The initial number of *S. sonnei* in all samples was 7 log CFU/mL, and then they were treated with four concentrations (0, 50, 100, 200 μM) of Stattic. The data are presented as the mean ± SD (n = 3, independent measurements).


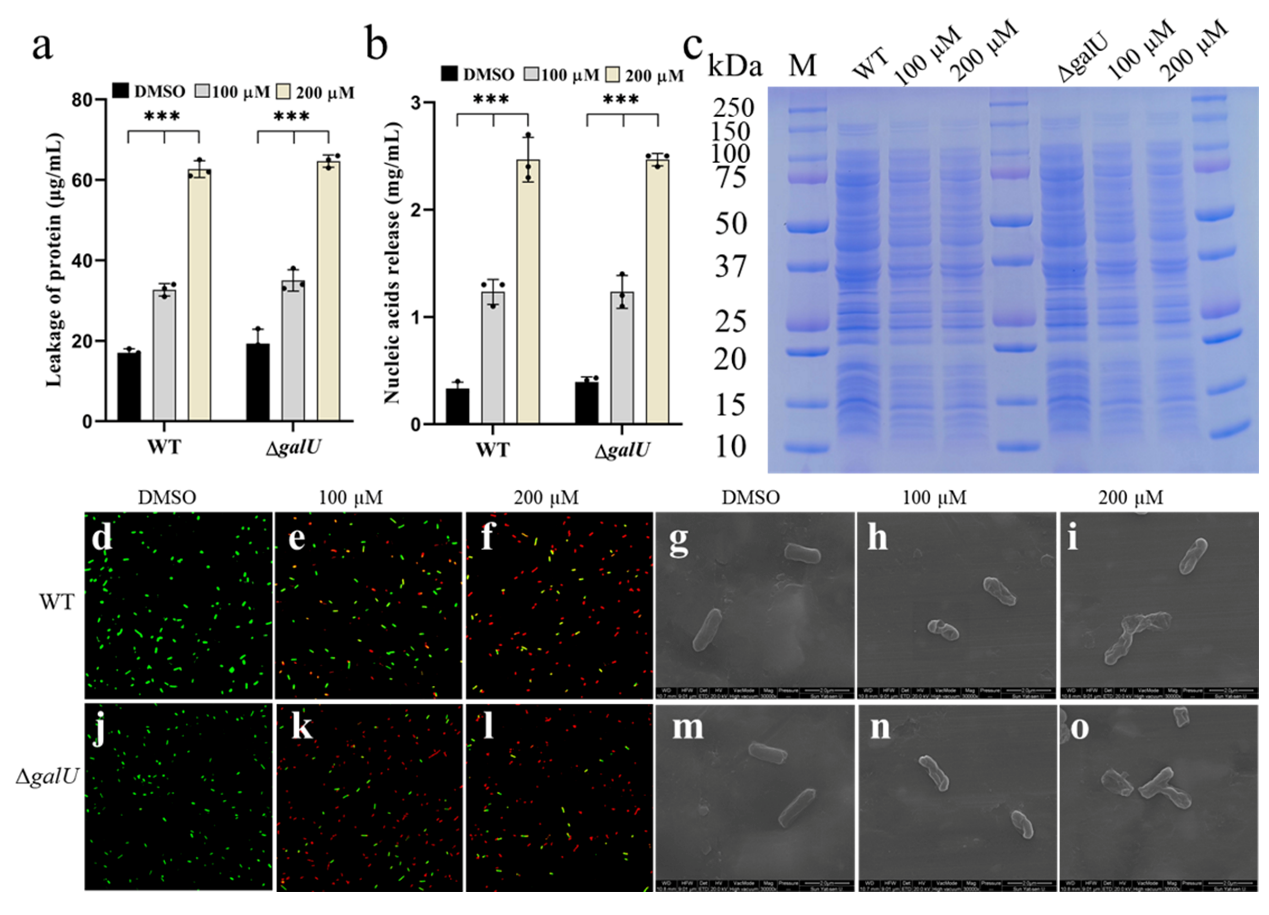


**Fig. s8** The sterilizing effect of Stattic. Leakage of proteins (a) and nucleic acids (b) from *S. sonnei* and *galU* mutant cells treated with Stattic (0, 100 µM, and 200 µM) for 10 h. (c) SDS‒PAGE profiles of *S. sonnei* and *galU* mutant intracellular proteins treated with Stattic at concentrations of 0, 100 µM, and 200 µM for 10 h. Lane M shows the protein band of the marker. Confocal laser scanning microscopy images of *S. sonnei* and *galU* mutant after exposure to Stattic at concentrations of 0 (d, j), 100 µM (e, k), or 200 µM (f, l). The bacteria with a complete membrane structure emit green fluorescence (SYTO 9), and the bacteria with damaged membrane structures emit red fluorescence (PI). The largest stimulation and launch wavelengths of the dyes of the two are 480/500 nm (SYTO 9) and 490/635 nm (PI), respectively. Scanning electron microscope images of *S. sonnei* and *galU* mutant after exposure to Stattic at concentrations of 0 (g, m), 100 µM (h, n), or 200 µM (i, o). The data are presented as the mean ± SD (n = 3, independent measurements).


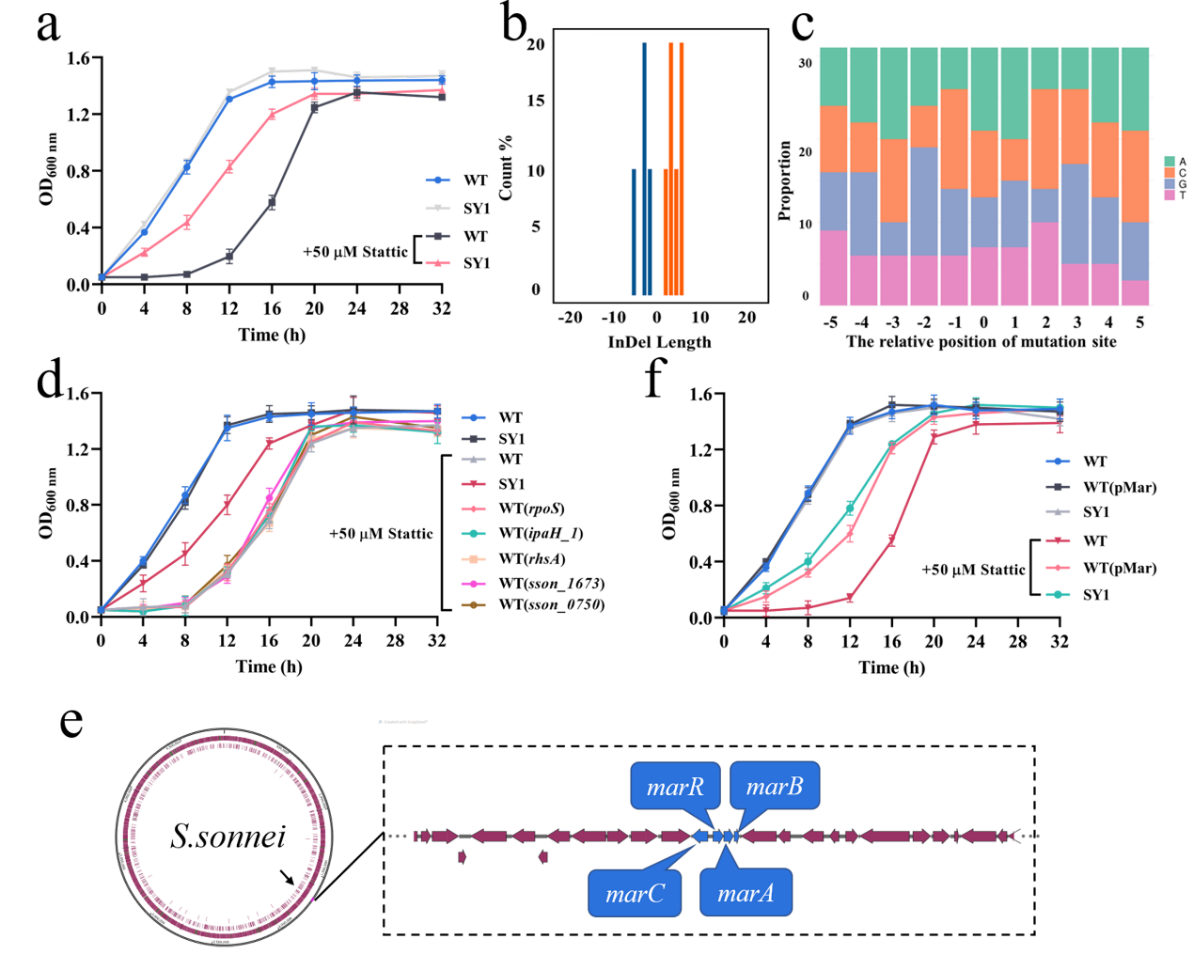


**Fig. s9** The mechanism of drug resistance of *S. sonnei* mutants by using whole-genome resequencing. (a) The growth curve of *S. sonnei* wild-type, SY1 and both in the presence of 50 μM Stattic. (b) The detection and statistics of indels. The abscissa is the length of insertion loss (+ is insertion, - is deletion), and the ordinate is the number of indels of the corresponding length. (c) Base preference statistics of SNP mutations. The abscissa is the base position before and after the mutation site, 0 is the SNP mutation site, the negative number represents the base before the mutation site, the positive number represents the base after the mutation site, and the ordinate is the corresponding number of different bases. (d) Growth curves of wild-type, SY1, WT(*rpoS*), WT(*ipaH_1*), WT(*rhsA*), WT(*sson_1673*), and WT(*sson_0750*) *S. sonnei* in the presence of 50 μM Stattic. (e) Location of Mar family genes on gene segments with variant copy numbers. (f) Growth curves of wild-type *S. sonnei*, SY1 and wild-type *S. sonnei in trans* with Mar family genes in the presence or absence of 50 μM Stattic. The data are presented as the mean ± SD (n = 3, independent measurements).


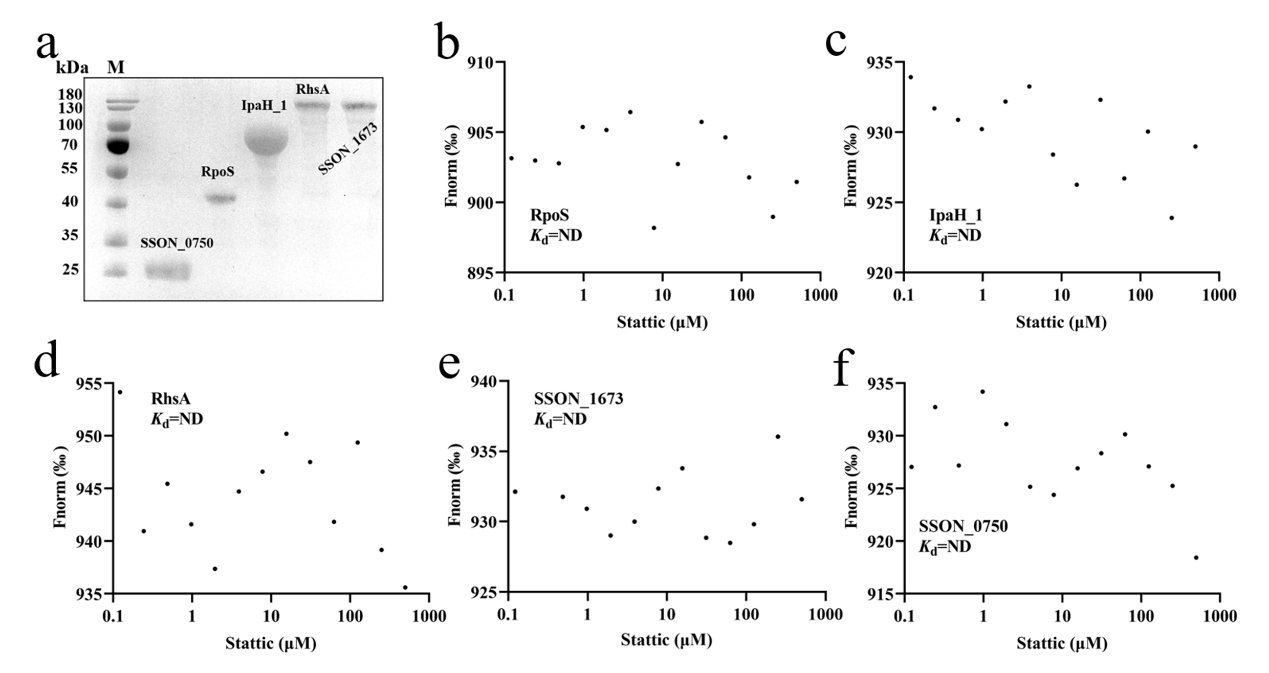


**Fig. s10** MST analysis of the binding of Stattic to potential germicidal targets. (a) SDS‒PAGE analysis of the five proteins (RpoS, RhsA, IpaH_1, SSON_0750, SSON_1673). (b-f) The detection of five proteins bound to Stattic by using MST. ND, not detected.

SUPPLEMENTARY TABLE 1 The predicted results of compounds binding to GalU using autodock.

| Compound | Structure | Binding Energy  (kcal/mol) | | Inhibit Constant  (µM) |
| --- | --- | --- | --- | --- |
| Carbadox | 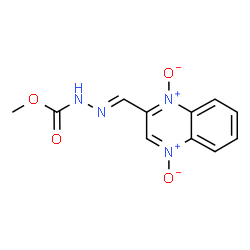 | -5.98 | 41.06 | |
| Panaxydol | 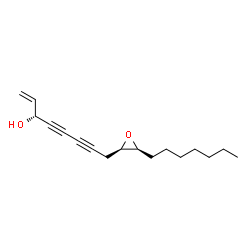 | -4.39 | 605.27 | |
| Brazilin | 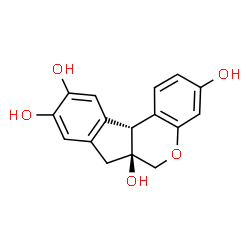 | -7.63 | 2.55 | |
| Pinostilbene | 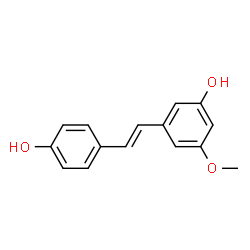 | -5.92 | 45.53 | |
| Ungeremine | 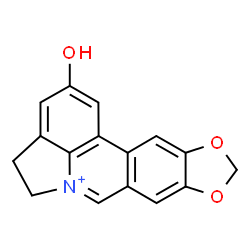 | -6.23 | 26.96 | |
| Stattic | 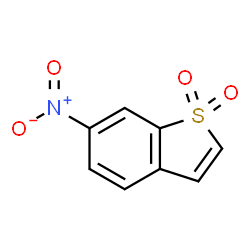 | -6.26 | 25.96 | |
| Bilirubin | 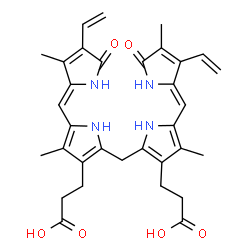 | -7.51 | 3.14 | |
| Thymoquinone | 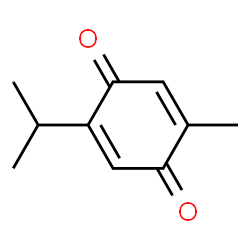 | -5.45 | 101.89 | |
| Ethyl gallate | 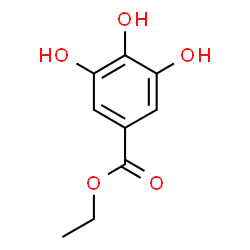 | -5.89 | 48.12 | |

SUPPLEMENTARY TABLE 2 Enzyme activity of GalU.

| **GalU protein** | **Activity (% WT)** | **Activity (% WT)**  **+ 10 µM Stattic** |
| --- | --- | --- |
| WT | 100% | 45% |
| R21A | ND | ND |
| K31A | 85% | 32% |
| K65A | 94% | 67% |
| Q109A | 65% | 63% |
| G179A | ND | ND |

SUPPLEMENTARY TABLE 3 The insertion or deletion mutations of SY1.

| **Mutation_type** | **Gene** | **Ref** | **Alt** |
| --- | --- | --- | --- |
| frameshift deletion | *sson_1673* | GG | - |
| frameshift insertion | *sson_1673* | - | AG |
| frameshift insertion | *rhsA* | - | TCCG |
| frameshift insertion | *rpoS* | - | CT |
| nonframeshift deletion | *sson_3814* | GTG | - |
| nonframeshift deletion | *rpoS* | CAT | - |
| nonframeshift insertion | *sson_3814* | - | TAC |
| Ref, reference; Alt, alter. | | | |

SUPPLEMENTARY TABLE 4 The SNV mutations of SY1.

| **Mutation_type** | **Gene** | **Ref** | **Alt** | **Site** |
| --- | --- | --- | --- | --- |
| nonsynonymous SNV | *sson_0750*. cds1: exon1: c. A367G: p., | A | G | N123D |
| nonsynonymous SNV | *ipaH_1*. cds1: exon1: c. G110A: p., | C | T | R37Q |
| nonsynonymous SNV | *ydcC*. cds1: exon1: c. G622A: p., | G | A | E208K |
| nonsynonymous SNV | *ydcC*. cds1: exon1: c. A638C: p., | A | C | K213T |
| nonsynonymous SNV | *sson_2652*. cds1: exon1: c. A289T: p., | T | A | S97C |
| nonsynonymous SNV | *sson_2679*. cds1: exon1: c. T500C: p., | A | G | I167T |
| synonymous SNV | *sson_0652*. cds1: exon1: c. T99C: p. G33G, | T | C |  |
| synonymous SNV | *ydcC*. cds1: exon1: c.C873T: p. T291T, | C | T |  |
| synonymous SNV | *sson_0750*. cds1: exon1: c.C363T: p. G121G, | C | T |  |
| synonymous SNV | *sson_0751*. cds1: exon1: c. G174A: p. A58A, | G | A |  |
| synonymous SNV | *ipaH_1*. cds1: exon1: c. A108G: p. P36P, | T | C |  |
| synonymous SNV | *sson_2666*. cds1: exon1: c. C1407T: p. D469D, | G | A |  |
| synonymous SNV | *sson_2666*. cds1: exon1: c.C1179T: p. Y393Y, | G | A |  |
| synonymous SNV | *sson_2679*. cds1: exon1: c. A240G: p. E80E, | T | C |  |
| synonymous SNV | *sson_2679*. cds1: exon1: c. C210T: p. I70I, | G | A |  |
| synonymous SNV | *sson_2784*. cds1: exon1: c.C189T: p. D63D, | G | A |  |
| synonymous SNV | *sson_2784*. cds1: exon1: c. A162G: p. T54T, | T | C |  |
| Ref, reference; Alt, alter. | | | | |

**SUPPLEMENTARY TABLE 5** Analysis of the homologs of GalU in various bacteria.

| **Bacteria** | | **Identity (%)** | **Accession No.** |
| --- | --- | --- | --- |
| *Acinetobacter* | |  |  |
|  | *A. albensis* | 58.22 | WP_193890123.1 |
|  | *A. baumannii* | 56.36 | WP_034705191.1 |
|  | *A. haemolyticus* | 56.66 | WP_161420069.1 |
|  | *A. indicus* | 57.73 | WP_104504113.1 |
|  | *A. johnsonii* | 58.42 | WP_125280574.1 |
|  | *A. junii* | 57.88 | HAK16805.1 |
|  | *A. schindleri* | 57.39 | WP_004895838.1 |
|  | *A. seohaensis* | 56.70 | GIT84415.1 |
|  | *A. terrae* | 58.36 | WP_067725770.1 |
|  | *A. terrestris* | 57.39 | WP_171537075.1 |
|  | *A. towneri* | 57.04 | WP_242770030.1 |
|  | *A. variabilis* | 57.39 | WP_125279476.1 |
|  | *A. venetianus* | 56.66 | WP_004882103.1 |
| *Aliivibrio* | |  |  |
|  | *A. finisterrensis* | 42.59 | WP_130044426.1 |
|  | *A. fischeri* | 43.54 | WP_065596661.1 |
|  | *A. logei* | 43.38 | WP_017022346.1 |
|  | *A. salmonicida* | 43.38 | WP_012549267.1 |
|  | *A. sifiae* | 43.75 | WP_060992574.1 |
|  | *A. wodanis* | 43.91 | CED72387.1 |
| *Azotobacter* | |  |  |
|  | *A. beijerinckii* | 42.59 | WP_090899810.1 |
|  | *A. chroococcum* | 42.96 | WP_089168206.1 |
|  | *A. salinestris* | 25.10 | WP_152386736.1 |
|  | *A. vinelandii* | 42.96 | WP_012701076.1 |
| *Buttiauxella* | |  |  |
|  | *B. agrestis* | 91.06 | WP_115629017.1 |
|  | *B. brennerae* | 90.73 | WP_064558005.1 |
|  | *B. ferragutiae* | 61.40 | WP_064545231.1 |
|  | *B. gaviniae* | 61.05 | WP_064512202.1 |
|  | *B. izardii* | 91.06 | WP_120063665.1 |
|  | *B. massiliensis* | 61.05 | WP_151994394.1 |
|  | *B. noackiae* | 91.72 | WP_034455847.1 |
|  | *B. warmboldiae* | 91.72 | WP_124024040.1 |
| *Cedecea* | |  |  |
|  | *C. colo* | 91.06 | WP_167607364.1 |
|  | *C. davisae* | 91.06 | WP_202308149.1 |
|  | *C. lapagei* | 94.04 | PKA31712.1 |
|  | *C. neteri* | 92.05 | WP_061278553.1 |
| *Citrobacter* | |  |  |
|  | *C. amalonaticus* | 98.01 | WP_044327862.1 |
|  | *C. braakii* | 59.86 | OXU10764.1 |
|  | *C. europaeus* | 96.35 | WP_213732991.1 |
|  | *C. farmeri* | 97.02 | WP_042318626.1 |
|  | *C. koseri* | 96.03 | WP_110494195.1 |
|  | *C. portucalensis* | 96.03 | WP_212025591.1 |
|  | *C. rodentium* | 97.35 | WP_012906012.1 |
|  | *C. sedlakii* | 96.69 | EJA2598364.1 |
|  | *C. telavivensis* | 97.35 | WP_152404327.1 |
|  | *C. youngae* | 96.36 | WP_006684279.1 |
| *Edwardsiella* | |  |  |
|  | *E. hoshinae* | 81.14 | WP_024524658.1 |
|  | *E. ictaluri* | 80.47 | WP_015870999.1 |
|  | *E. piscicida* | 80.47 | WP_226086682.1 |
|  | *E. tarda* | 81.82 | WP_035604915.1 |
| *Enterobacter* | |  |  |
|  | *E. asburiae* | 94.37 | WP_181593282.1 |
|  | *E. bugandensis* | 94.37 | WP_232928214.1 |
|  | *E. cancerogenus* | 94.35 | WP_137273318.1 |
|  | *E. cloacae* | 96.36 | SAD31485.1 |
|  | *E. hormaechei* | 99.67 | QLW06381.1 |
|  | *E. huaxiensis* | 93.71 | WP_119937691.1 |
|  | *E. kobei* | 94.37 | WP_045268994.1 |
|  | *E. mori* | 94.70 | WP_211445427.1 |
|  | *E. oligotrophicus* | 94.35 | WP_152081321.1 |
|  | *E. quasimori* | 94.37 | WP_126545985.1 |
|  | *E. quasiroggenkampii* | 94.37 | WP_252073170.1 |
|  | *E. roggenkampii* | 94.70 | HJD72012.1 |
|  | *E. wuhouensis* | 94.37 | WP_131632388.1 |
| *Franconibacter* | |  |  |
|  | *F. helveticus* | 92.38 | WP_024553055.1 |
|  | *F. pulveris* | 92.05 | WP_029591513.1 |
| *Pseudomonas* | |  |  |
|  | *P. abyssi* | 56.51 | WP_096004168.1 |
|  | *P. aeruginosa* | 45.89 | RRJ53230.1 |
|  | *P. anguilliseptica* | 42.96 | WP_090386263.1 |
|  | *P. argentinensis* | 44.44 | WP_070887336.1 |
|  | *P. composti* | 43.33 | WP_061239366.1 |
|  | *P. fluorescens* | 43.92 | PZP39410.1 |
|  | *P. guguanensis* | 42.96 | WP_090432453.1 |
|  | *P. jilinensis* | 59.93 | WP_119701853.1 |
|  | *P. laoshanensis* | 61.38 | WP_096347511.1 |
|  | *P. mendocina* | 43.33 | WP_106741094.1 |
|  | *P. mosselii* | 100.00 | WP_250159398.1 |
|  | *P. nanhaiensis* | 56.12 | WP_223655249.1 |
|  | *P. oleovorans* | 42.96 | WP_179576505.1 |
|  | *P. phragmitis* | 59.93 | WP_080050160.1 |
|  | *P. punonensis* | 43.70 | WP_073266974.1 |
|  | *P. reactans* | 73.70 | NWA39830.1 |
|  | *P. saliphila* | 57.88 | WP_150306224.1 |
|  | *P. saudimassiliensis* | 60.34 | WP_044500706.1 |
|  | *P. sediminis* | 42.96 | WP_179544834.1 |
|  | *P. wenzhouensis* | 42.96 | WP_230924460.1 |
|  | *P. xionganensis* | 43.33 | WP_160345448.1 |
|  | *P. yangmingensis* | 59.79 | WP_093476987.1 |
| *Klebsiella* | |  |  |
|  | *K. aerogenes* | 93.69 | BCZ62438.1 |
|  | *K. michiganensis* | 93.92 | HCE8855697.1 |
|  | *K. oxytoca* | 93.92 | HBM3141561.1 |
|  | *K. pasteurii* | 93.92 | WP_142983099.1 |
|  | *K. pneumoniae* | 94.37 | SSW79447.1 |
|  | *K. quasipneumoniae* | 94.92 | WP_153678692.1 |
|  | *K. quasivariicola* | 94.92 | WP_224421552.1 |
| *Kluyvera* | |  |  |
|  | *K. ascorbata* | 93.38 | WP_249023192.1 |
|  | *K. cryocrescens* | 93.05 | WP_061281375.1 |
|  | *K. georgiana* | 93.38 | WP_065357183.1 |
|  | *K. intermedia* | 92.05 | WP_234508863.1 |
|  | *K. sichuanensis* | 60.21 | WP_185666996.1 |
| *Kosakonia* | |  |  |
|  | *K. arachidis* | 92.38 | WP_090120613.1 |
|  | *K. cowanii* | 91.39 | WP_242389082.1 |
|  | *K. oryzae* | 92.72 | WP_064566027.1 |
|  | *K. oryzendophytica* | 92.72 | WP_061496262.1 |
|  | *K. oryziphila* | 92.38 | WP_090138642.1 |
|  | *K. pseudosacchari* | 93.05 | WP_086868643.1 |
|  | *K. quasisacchari* | 92.72 | WP_131409366.1 |
|  | *K. radicincitans* | 93.05 | WP_043953465.1 |
|  | *K. sacchari* | 93.05 | WP_071908159.1 |
| *Leclercia* | |  |  |
|  | *L. adecarboxylata* | 94.37 | KML21735.1 |
|  | *L. pneumoniae* | 59.25 | WP_207292046.1 |
| *Lelliottia* | |  |  |
|  | *L. amnigena* | 94.70 | WP_202667859.1 |
|  | *L. aquatilis* | 24.66 | WP_103946176.1 |
|  | *L. jeotgali* | 94.04 | ASV55712.1 |
|  | *L. nimipressuralis* | 94.37 | OIR50021.1 |
| *Listeria* | |  |  |
|  | *L. booriae* | 43.15 | WP_185400089.1 |
|  | *L. cossartiae* | 42.81 | WP_185587329.1 |
|  | *L. farberi* | 42.65 | WP_185318469.1 |
|  | *L. fleischmannii* | 42.09 | WP_007472744.1 |
|  | *L. grandensis* | 41.78 | WP_187136217.1 |
|  | *L. grayi* | 40.96 | WP_040486401.1 |
|  | *L. ilorinensis* | 42.03 | WP_239256438.1 |
|  | *L. immobilis* | 43.37 | WP_185349218.1 |
|  | *L. innocua* | 42.47 | WP_187121484.1 |
|  | *L. ivanovii* | 43.73 | WP_200372520.1 |
|  | *L. marthii* | 42.81 | WP_185531666.1 |
|  | *L. monocytogenes* | 45.02 | HAC3243914.1 |
|  | *L. riparia* | 43.49 | WP_036098993.1 |
|  | *L. rocourtiae* | 42.81 | WP_036070061.1 |
|  | *L. rustica* | 43.49 | WP_181677569.1 |
|  | *L. seeligeri* | 42.12 | WP_185478045.1 |
|  | *L. valentina* | 41.36 | WP_167628123.1 |
|  | *L. weihenstephanensis* | 43.73 | WP_036063394.1 |
|  | *L. welshimeri* | 42.12 | WP_185325340.1 |
| *Mangrovibacter* | |  |  |
|  | *M. phragmitis* | 91.39 | WP_064593969.1 |
|  | *M. plantisponsor* | 66.10 | WP_110027002.1 |
|  | *M. yixingensis* | 91.06 | WP_226570547.1 |
| *Photobacterium* | |  |  |
|  | *P. alginatilyticum* | 76.53 | WP_160648654.1 |
|  | *P. angustum* | 75.17 | WP_045151996.1 |
|  | *P. aquae* | 75.09 | WP_047880108.1 |
|  | *P. atrarenae* | 75.25 | WP_255388152.1 |
|  | *P. chitinilyticum* | 75.85 | WP_128781967.1 |
|  | *P. damselae* | 76.27 | WP_237041418.1 |
|  | *P. gaetbulicola* | 74.66 | WP_039467634.1 |
|  | *P. galatheae* | 76.11 | WP_250684220.1 |
|  | *P. ganghwense* | 75.77 | WP_047887554.1 |
|  | *P. halotolerans* | 75.43 | WP_046218789.1 |
|  | *P. indicum* | 75.60 | WP_107253962.1 |
|  | *P. leiognathi* | 75.51 | WP_107235158.1 |
|  | *P. lipolyticum* | 76.29 | WP_107284339.1 |
|  | *P. lutimaris* | 74.42 | WP_107349416.1 |
|  | *P. marinum* | 76.79 | WP_007470110.1 |
|  | *P. profundum* | 74.83 | PSV60693.1 |
|  | *P. proteolyticum* | 76.87 | WP_075764142.1 |
|  | *P. rosenbergii* | 75.08 | WP_222550115.1 |
|  | *P. salinisoli* | 76.71 | WP_120511419.1 |
|  | *P. sanctipauli* | 75.60 | WP_107271575.1 |
| *Phytobacter* | |  |  |
|  | *P. diazotrophicus* | 66.67 | WP_110296278.1 |
|  | *P. massiliensis* | 93.71 | WP_044174876.1 |
|  | *P. palmae* | 92.05 | SFE52985.1 |
|  | *P. sp. SCO41* | 65.99 | WP_172960920.1 |
|  | *P. ursingii* | 92.38 | VTP14173.1 |
| *Raoultella* | |  |  |
|  | *R. electrica* | 59.15 | WP_141964241.1 |
|  | *R. ornithinolytica* | 93.19 | VTN42001.1 |
|  | *R. planticola* | 60.00 | BAF47039.1 |
|  | *R. terrigena* | 93.58 | WP_076947657.1 |
| *Salinivibrio* | |  |  |
|  | *S. costicola* | 39.16 | WP_077669459.1 |
|  | *S. kushneri* | 39.58 | WP_077649953.1 |
|  | *S. proteolyticus* | 39.36 | WP_077675882.1 |
|  | *S. sharmensis* | 38.81 | WP_077772660.1 |
|  | *S. siamensis* | 36.67 | WP_077668722.1 |
|  | *S. socompensis* | 39.58 | WP_025673824.1 |
| *Salmonella* | |  |  |
|  | *S. enterica* | 97.35 | WP_080176773.1 |
|  | *S. sphingomonas* |  |  |
|  | *S. sphingomonas elodea* | 41.16 | WP_010545383.1 |
| *Vibrio* | |  |  |
|  | *V. alginolyticus* | 100.00 | MCA6722917.1 |
|  | *V. chagasii* | 43.17 | CAH7427434.1 |
|  | *V. cholerae* | 43.75 | MUK01734.1 |
|  | *V. cidicii* | 42.59 | WP_061900353.1 |
|  | *V. cincinnatiensis* | 41.85 | WP_238130498.1 |
|  | *V. cyclitrophicus* | 70.49 | KAA8597027.1 |
|  | *V. fluvialis* | 41.85 | MBY8309000.1 |
|  | *V. hyugaensis* | 69.44 | WP_045499814.1 |
|  | *V. jasicida* | 68.75 | WP_045407531.1 |
|  | *V. lentus* | 43.17 | WP_102284862.1 |
|  | *V. maritimus* | 59.46 | GAL37058.1 |
|  | *V. metschnikovii* | 41.08 | WP_217524980.1 |
|  | *V. mimicus* | 42.96 | WP_005511057.1 |
|  | *V. natriegens* | 41.48 | WP_176290756.1 |
|  | *V. owensii* | 66.90 | WP_039987528.1 |
|  | *V. paracholerae* | 42.96 | WP_162892021.1 |
|  | *V. parahaemolyticus* | 98.01 | KKF70462.1 |
|  | *V. splendidus* | 41.48 | WP_102442506.1 |
|  | *V. stylophorae* | 73.88 | WP_237465126.1 |
|  | *V. tarriae* | 42.96 | ASK56375.1 |
|  | *V. vulnificus* | 76.43 | HAS8350312.1 |
| *Yersinia* | |  |  |
|  | *Y. aldovae* | 74.92 | WP_145521599.1 |
|  | *Y. aleksiciae* | 75.17 | WP_048620153.1 |
|  | *Y. alsatica* | 75.26 | WP_186372862.1 |
|  | *Y. artesiana* | 74.92 | WP_174851136.1 |
|  | *Y. bercovieri* | 75.34 | PHZ28457.1 |
|  | *Y. canariae* | 75.25 | WP_145556284.1 |
|  | *Y. enterocolitica* | 75.95 | WP_042661472.1 |
|  | *Y. frederiksenii* | 75.34 | OVZ99223.1 |
|  | *Y. intermedia* | 75.95 | WP_186378475.1 |
|  | *Y. kristensenii* | 75.09 | WP_100191246.1 |
|  | *Y. mollaretii* | 74.83 | WP_049646654.1 |
|  | *Y. nurmii* | 74.91 | WP_049596592.1 |
|  | *Y. rohdei* | 74.58 | WP_004713539.1 |
|  | *Y. ruckeri* | 75.60 | WP_234049353.1 |
|  | *Y. thracica* | 74.49 | WP_050114310.1 |
